# The effects of d-aspartic acid supplementation in resistance-trained men over a three month training period: A randomised controlled trial

**DOI:** 10.1371/journal.pone.0182630

**Published:** 2017-08-25

**Authors:** Geoffrey W. Melville, Jason C. Siegler, Paul W. M. Marshall

**Affiliations:** School of Science and Health, Western Sydney University, Sydney, Australia; Texas A&M University, UNITED STATES

## Abstract

**Context:**

Research on d-aspartic acid (DAA) has demonstrated increases in total testosterone levels in untrained men, however research in resistance-trained men demonstrated no changes, and reductions in testosterone levels. The long-term consequences of DAA in a resistance trained population are currently unknown.

**Objective:**

To evaluate the effectiveness of DAA to alter basal testosterone levels over 3 months of resistance training in resistance-trained men.

**Design:**

Randomised, double-blind, placebo controlled trial in healthy resistance-trained men, aged 18–36, had been performing regular resistance training exercise for at least 3 d.w^-1^ for the previous 2 years. Randomised participants were 22 men (d-aspartic acid n = 11; placebo n = 11) (age, 23.8±4.9 y, training age, 3.2±1.5 y).

**Intervention:**

D-aspartic acid (6 g.d^-1^, DAA) versus equal-weight, visually-matched placebo (PLA). All participants performed 12 weeks of supervised, periodised resistance training (4 d.w^-1^), with a program focusing on all muscle groups.

**Measures:**

Basal hormones, total testosterone (TT), free testosterone (FT), estradiol (E_2_), sex-hormone-binding globulin (SHBG) and albumin (ALB); isometric strength; calf muscle cross-sectional area (CSA); calf muscle thickness; quadriceps muscle CSA; quadriceps muscle thickness; evoked V-wave and H-reflexes, were assessed at weeks zero (T1), after six weeks (T2) and after 12 weeks (T3).

**Results:**

No change in basal TT or FT were observed after the intervention. DAA supplementation (n = 10) led to a 16%, 95% CI [-27%, -5%] reduction in E_2_ from T1-T3 (p<0.01). The placebo group (n = 9) demonstrated improvements in spinal responsiveness (gastrocnemius) at the level of the alpha motoneuron. Both groups exhibited increases in isometric strength of the plantar flexors by 17%, 95% CI [7%, 28%] (p<0.05) as well as similar increases in hypertrophy in the quadriceps and calf muscles.

**Conclusions:**

The results of this paper indicate that DAA supplementation is ineffective at changing testosterone levels, or positively affecting training outcomes. Reductions in estradiol and the blunting of peripheral excitability appear unrelated to improvements from resistance training.

**Trial registration:**

Australian New Zealand Clinical Trials Registry ACTRN12617000041358

## Introduction

D-aspartic acid (DAA) is an amino acid that exists in central nervous and reproductive tissues. Animal and human research suggest that DAA functions in the development of the nervous system as well as hormonal regulation [1, 2]. Data in mammalian studies indicate that DAA supplementation can influence the hypothalamic-pituitary-gonadal axis (HPG) at the level of the hypothalamus [3], anterior pituitary [3, 4] and the testes [4, 5]. Accumulation of DAA at these sites is associated with an upregulation of testosterone production in these animals, as well as upstream effectors of the HPG axis (i.e. luteinizing hormone).

Early human research demonstrated that three grams per day of DAA supplementation increased total testosterone in untrained men by ~42% [6, 7]. Subsequently the popularity of DAA supplementation in recreational resistance training has increased, owing to the positive relationship between testosterone levels and mechanisms of muscular hypertrophy, such as increased protein synthesis [8, 9] and satellite cell proliferation [10]. However in resistance-trained men, three grams of DAA per day resulted in no meaningful change in testosterone levels, or training outcomes after 14 [11] or 30 days of supplementation [12]. Indeed, Melville and colleagues reported that a larger daily dose of DAA (6 g.d^-1^) actually decreased basal testosterone by ~12.5% after 14 days of supplementation, suggesting a deleterious effect on negative feedback mechanisms of the HPG axis [11]. This reduction is a particularly concerning finding considering the supposed importance of basal testosterone, with respect to hypertrophic training outcomes. It is unclear if the observed decline in basal testosterone after 14 days will continue if supplementation is continued for a longer timeframe (i.e. three months). Moreover, the relationship to training outcomes if testosterone continues to decline, or otherwise is maintained at a new basal level, is unknown.

The potential adverse effects of DAA on testosterone, in addition to affecting hypertrophic outcomes, may also have an effect on the neural mechanisms associated with increased muscular strength. Strength appears to be a function of both the size [13] and composition of muscle [14], in addition to the rate and magnitude of output from the nervous system [15, 16]. Resistance training increases the voluntary activation of the nervous system [17], in addition to increasing the input-output response of cells in the spinal cord [18–20]. There is a growing body of research that suggests DAA fills the criteria of a neurotransmitter [21–23], which potentially could provide strength improvements via increased neurotransmitter availability. Neural plasticity, which is observed in untrained individuals is a key explanatory mechanism of strength development [24], and can involve cells at the spinal and supraspinal level. Measures of neural plasticity that examine spinal and supraspinal output, such as the H-reflex and V-wave respectively, have not been measured following a period of resistance exercise in trained individuals and as such, it is not clear if trained populations have the same neural adaptation response to resistance training as untrained individuals. The link between testosterone and changes in the corticospinal pathway has been explored in humans with artificially induced testosterone levels [25]. These increases in testosterone were concurrent with reduced threshold of the evoked potentials from transcranial magnetic stimulation (TMS), indicating that testosterone can positively increase the output from a given input, within the corticospinal pathway [25]. It is plausible that artificially changing testosterone levels, via supplementation may affect strength or power, by altering the efficiency of the corticospinal pathway. The effects of DAA on neural plasticity has yet to be researched in humans.

The primary objective of this study was to evaluate the effectiveness of DAA to alter basal testosterone levels over three months of resistance training. A secondary objective was to establish potential mechanisms for changes in strength and hypertrophy. Based on our previous findings, it was hypothesised that the DAA group would experience decreased total testosterone and free testosterone. In addition, it was hypothesised that the DAA group would experience decreased strength, which would be explained by changes in the corticospinal pathway.

## Methods

### Subjects

**Fig 1:**
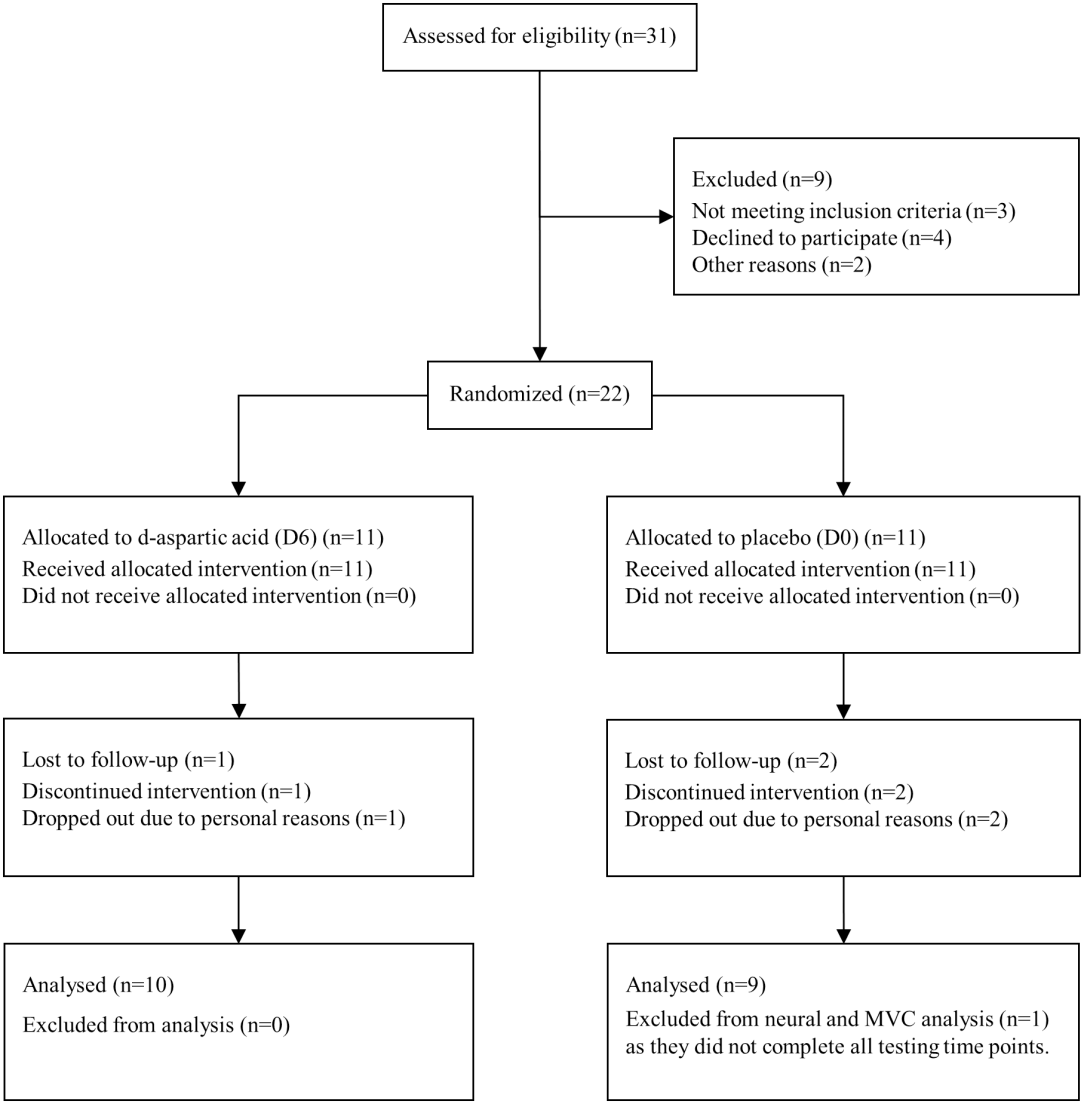
Consort flow diagram.

The present study was approved by the Western Sydney University Research Ethics Committee (H10087) and data are ethically restricted for reasons relating to participant privacy. All relevant data are contained within the paper and supporting files. All participants gave written consent and completed a medical history check. The study was carried out by the Declaration of Helsinki. Participants were recruited from the University campus via flyers, lecture announcements and online intranet advertising. In addition, recruitment from the western Sydney area was promoted via Facebook advertising. In response to recruitment 31 people were assessed for eligibility. Twenty-two subjects were recruited, three dropped out due to personal reasons, leaving 19 that completed the study (Fig 1). A rolling recruitment strategy was conducted from 17^th^ of March to the 16^th^ of December 2014, with participants beginning soon after they were recruited, thus participants began the study at different time periods. Final testing was conducted at the end of the 12 week training period and the trial was ended according to a set schedule for each participant. The last day of data collection was the 10^th^ March 2015. The sample size for the present study was determined from the reduction in free testosterone observed in previous DAA research [11]. Free testosterone changes in the six-gram group demonstrated an effect size of 1.17, which required 12 participants to be adequately powered. A Means statistical test (matched pairs) using G Power v 3.1. was used to estimate effect size using group parameters from the previous research [11]. Power calculations were determined A Priori with an alpha level of 0.05 and two tailed significance. Participant demographics are presented in Table 1.

**Table 1:** Comparison of participant characteristics for 6 g/d of d-aspartic acid (DAA) and placebo (PLA), presented as mean±SD.

	PLA	DAA
	N = 9	N = 10
Age (years)	25.4 ± 6.4	22.4 ± 2.6
Training age (years)	3.3 ± 1.7	3.1 ± 1.3
Height (m)	178.3 ± 6.2	180.4 ± 6.4
Body Mass (kg)	82.5 ± 9.0	80.5 ± 10.2

*Inclusion Criteria*: aged 18–36; have no acute or chronic medical conditions; have the ability to bench press 100% bodyweight; had been performing regular resistance training exercise for at least three days per week for the previous two years; and not supplementing diet with any ergogenic or testosterone boosting supplements.

### Study design

The present study was a randomised, double-blinded, and placebo-controlled research design. Subjects attended three laboratory sessions, located at the University campus, baseline testing (T1), midpoint testing (T2) and post testing (T3). These testing sessions involved: fasted blood draws for serum hormonal analysis; Brightness-mode (B-mode) ultrasound of the quadriceps and calf muscles for hypertrophy changes; electrical stimulation of the tibial nerve to determine H-reflex and V-wave responses of the plantar flexors; and isometric maximal voluntary contraction (MVC) of the plantar flexor muscles. Following T1, all participants followed a structured resistance training protocol, which was combined with their daily supplementation protocol. Participants were assigned to one of two experimental groups: placebo (PLA, 6g rice flour, n = 11) or d-aspartic acid (6g DAA, n = 11). Three participants were lost to follow-up, thus analysed participants included PLA, n = 9; DAA, n = 10. One participant from the placebo group did not have sufficient data for plantar flexor strength and neural measures, thus was omitted from those analyses. All participants consumed 10 identical opaque capsules each morning with water before they had breakfast for 12 weeks. Adherence to daily supplementation was controlled for via prompts at supervised training sessions, in addition the number of pills left were compared to their set schedule each week. Participants were randomly allocated to treatment groups following a block randomisation procedure (block size of four) based on a computer-generated list of random numbers. Group allocation was managed by a technical officer, while the primary investigator and participants was kept blind to group assignment throughout the experimental intervention, and data analysis. The protocol as explained in the original participant information sheet, included 1RM testing for the squat and bench press. These were omitted due to time constraints of participants on testing days, as well as the fact that isometric testing would provide a stronger match with neural testing of the plantar flexors. As this study utilised a commercially available supplement rather than a drug, it was not expected that this study would fit the definition of a clinical trial, therefore the clinical registration of this study was performed retrospectively (ANZCTR).

### Training protocol

Participants trained four days per week, with supervised sessions conducted a minimum of once per week. The participants training was monitored using training diaries. The prescribed training involved five exercises that included 3–5 sets of various repetition maximum (RM) prescriptions (2RM– 10RM). A primary lift was prescribed for each day–deadlift, bench press, good morning/stiff-leg deadlift and squat, with accessory exercises for balance and volume. These accessory exercises included: seated rows, pull-down, bent over row, biceps curl, chin-ups, bench press, overhead press, pec fly, dip and back extension. See Table 2 for programming of measurement specific exercises.

**Table 2:** Program design for exercises that primarily targeted the quadriceps and calf muscles. Prescribed training was either repetition maximum (RM) or body weight to failure (BWF).

Weeks 1–6	Set 1	Set 2	Set 3	Set 4	Set 5
Day 1					
Split squat	10RM	8RM	6RM		
1-leg calf raise	BWF	BWF	BWF		
Day 3					
Seated calf	10RM	10RM	8RM	8RM	6RM
Day 4					
Front squat	8RM	6RM	5RM		
Leg press	8RM	6RM	5RM		
Leg extension	10RM	10RM	10RM	10RM	
Step-ups	10RM	10RM	10RM		
Weeks 7–12	Set 1	Set 2	Set 3	Set 4	Set 5
Day 1					
Bulgarian split squat	8RM	6RM	6RM		
1-leg calf raise	BWF	BWF	BWF		
Day 3					
Seated calf	10RM	8RM	8RM	6RM	
Day 4					
Back squat	8RM	8RM	6RM	6RM	
Leg press	10RM	6RM	8RM	6RM	6RM
Leg extension	10RM	10RM	10RM	8RM	
Step-ups	5RM	5RM	5RM	5RM	

Periodisation changes were implemented at week six to improve motivation and prevent overtraining. These changes can be observed in Table 2, with an increase in training intensity with some of the exercises, but they did not affect the overall layout of the protocol. All participants were asked to perform 48 sessions in the gym in total (average number of sessions performed 47.5±0.8, an adherence rate of 99.0%).

### Testing sessions

**Fig 2:**
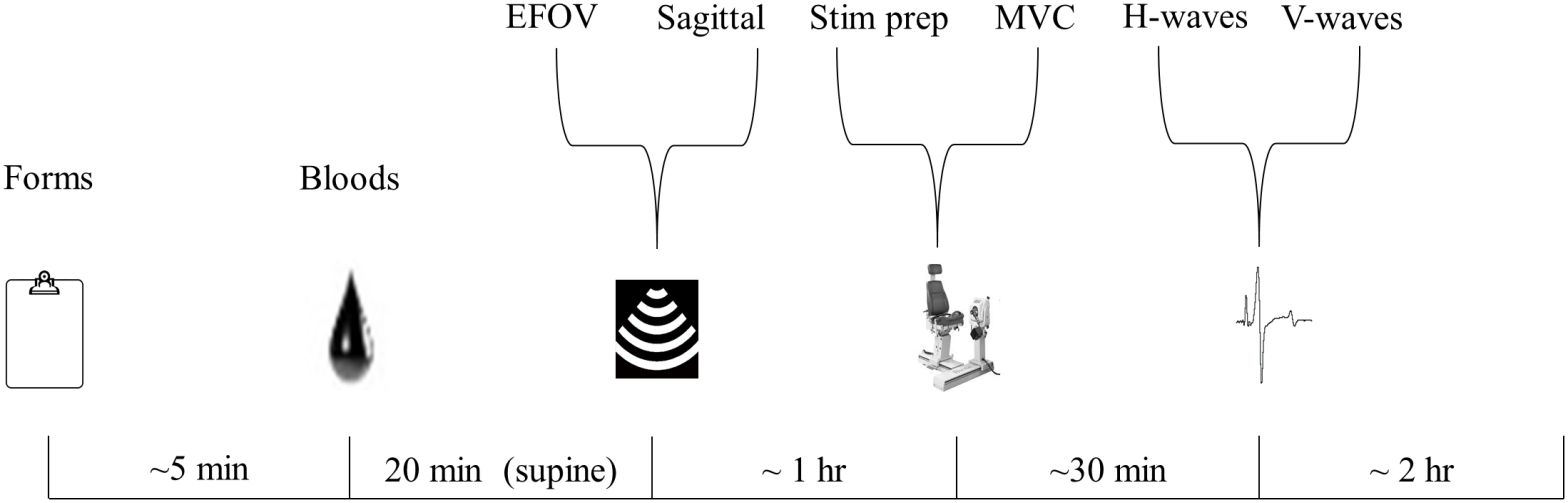
Testing procedures overview. In chronological order, the study protocol comprised of: Consent and forms; venepuncture blood draws; ultrasound testing of the quadriceps and calf muscles; EMG and stimulation preparation; isometric MVCs (plantar flexors); and determination of H-wave curve and V-waves via electrical stimulation.

During laboratory sessions, the subject was asked to lie down upon arrival. After checking for pre-testing adherence protocols (fasting status) participants were asked to lie down for two minutes. Blood was then extracted from the antecubital vein via venepuncture. Participants remained lying down for 20 minutes before ultrasound procedures followed. Following ultrasound testing, the subject was seated into an isokinetic dynamometer (KinCom 125, version 5.32, Chattanooga, TN, USA). After the KinCom was configured, the subject was prepared for EMG and the cathode location was determined for transcutaneous nerve stimulation. The protocol for the determination of isometric plantar flexor strength, H-wave, M_max_ and V-waves followed. The overview of the study protocol is visually represented in Fig 2.

### Fasted blood draws

Participants were instructed to fast for 12-hours and avoid strenuous exercise and alcohol consumption the day before testing. For each participant, the initial blood draw was scheduled in the morning between 7:00–10:00, with subsequent draws time-matched on an individual basis. Whole blood was collected using serum separator tubes (SST^™^ II Advance, BD Vacutainer^®^), and rested for 45 minutes in an air-conditioned room (19°C). Collection tubes were centrifuged using a fixed angle rotor centrifuge (ADAMS^®^ Compact II Centrifuge, V:227, Becton Dickinson & Co), run for 15 minutes at 2700 rpm and 828 x g. Serum was aliquoted and stored at ‒80°C until analysis by an external laboratory (Douglas Hanly Moir Pathology, Macquarie Park, NSW, Australia). A single analysis of serum was performed for total testosterone (TT), estradiol (E_2_), sex-hormone-binding-globulin (SHBG) and albumin (ALB). Testosterone and SHBG were measured via electrochemiluminescence immunoassay, on a Roche E170 system (Roche Diagnostics, Dee Why, NSW, Australia), with a limit of detection values of 0.025 ng/ml and 0.350 nmol/l respectively. Albumin was measured via bromocresol green succinate buffer method (BCG), on an Abbott ARCHITECT c16000 (Abbott Laboratories, Abbott Park, Illinois, USA). The limit of detection of the ALB assay is 30 g/l. Estradiol was measured via chemiluminescent microparticle immunoassay, on an Abbott i2000 (Abbott Laboratories, Abbott Park, Illinois, USA) and the analytical sensitivity of this assay is ≤ 10 pg/ml. Free testosterone (FT) was calculated from TT, SHBG and ALB [26].

### Ultrasound

Participants were asked to rest in a supine position for 20 minutes, to allow for the fluid shift in the muscles to stabilise [27]. Extended-field-of-view (EFOV) images were conducted at the inferior third of the quadriceps muscles, measured as a third of the distance between the centre of the knee joint and the Anterior Superior Iliac Spine (ASIS). B-mode ultrasound imaging was conducted using an Echo Blaster 128 family scanner and Echo Wave II v2.3.6 (Telemed, Vilnius, Lithuania) software. Quality control methodology has been previously outlined [28]. These images were analysed for Cross Sectional Area (CSA) of the quadriceps muscles; vastus lateralis (VL), vastus intermedialis (VI), vastus medialis (VM) and rectus femoris (RF). A minimum of three images was obtained for each time point, with the average values from these images used for data analysis. Images were analysed using ImageJ 1.46 (National Institutes of Health, Bethesda, Maryland, USA) public domain software package. Intra-experimenter reliability (CV) of the EFOV method was 2.26%.

Muscle thickness was determined for VL and VI at the inferior third (33%) and the midpoint of the quadriceps (50%). To obtain sagittal images the probe was manipulated until the superficial, and deep aponeuroses were parallel, and the pennation fibres were straight rather than curved. Muscle thickness was obtained for vastus lateralis (VL_33%,_ VL_50%_) and vastus intermedialis (VI_33%,_ VI_50%_). Pennation angle for vastus lateralis (VL_Angle_) was determined at the midpoint quadriceps site (50% between the centre of the knee joint and ASIS). To determine calf muscle thickness, the distance between the lateral malleolus and the fibular head was divided into three sites: Superior quarter, SOL_75%_ and GAS_75%_; superior third, SOL_67%_ and GAS_67%_; and midpoint, SOL_50%_ and GAS_50%_. Intra-experimenter reliability (CV) of the analysis of the sagittal images was 2.99% for the quadriceps thickness, 8.68% for calf thickness and 13.67% for pennation angle.

### EMG

Electromyography was recorded from the soleus and gastrocnemius muscles using bipolar Ag/AgCl electrodes (Maxensor; Medimax Global, Australia). The skin was prepared following standardised procedure to reduce impedance below five kΩ. Soleus electrodes were placed at two-thirds of the distance between the medial condyle of the femur and the medial malleolus. Two electrodes were placed on the muscle belly for the medial gastrocnemius site, and the medial malleolus landmark was used for the reference electrode. Placement of the electrodes was recorded to ensure consistency between testing sessions. A ML138 Octal BioAmp (common mode rejection ratio >85 dB @ 50 Hz, input impedance 200 MΩ) with 16-bit analog-to-digital conversion, sampled at 2 kHz (ADI Instruments, Sydney, NSW, Australia) was used to record sEMG signals into LabChart v7.3.7. Raw signals were filtered with a fourth-order Bessel filter between 20–500 Hz.

### Isometric force

Isometric testing was chosen over dynamic 1RM strength testing, as it was believed to be a stronger and more reliable match to the neural protocol. The isokinetic dynamometer (KinCom 125, version 5.32, Chattanooga, TN, USA) was configured, so the seat was set upright, with the participant’s hip and knee flexed to 90° degrees and the subject’s lateral malleolus in line with the centre of rotation of the lever arm. The seat angle was adjusted so that there was no gap between the knee and the edge of the seat to prevent unwanted muscle activity. The participants left foot was attached to the plantar-flexion–dorsiflexion attachment with hook-and-loop straps. Two seatbelts were applied across the thighs and chest. Subjects were warmed up with three submaximal contractions, ~50%, ~75%, ~90% Maximal Voluntary Contraction (MVC) spaced one minute apart. After sufficient warm up, three MVCs were recorded, with two minutes rest given between each MVC. Subjects were instructed, from complete relaxation, to contract their plantar flexors as hard and as fast as possible, holding the contraction for five seconds. Verbal encouragement was given during MVC attempts. This was standardised by repeating the word “GO” during the five seconds of contraction. Visual feedback of the instantaneous torque production was displayed on a screen that participants could see. Torque output signals were directly sampled from the dynamometer at 2 kHz (PowerLab, ADInstruments, Sydney, NSW, Australia). MVC was defined as the peak isometric torque (N.m) exerted within the 5s force trace.

### Posterior tibial nerve stimulation

H-reflexes, M-waves and V-waves were evoked from the soleus and gastrocnemius using a 1-ms square wave pulse, delivered by a constant current stimulator (DS7AH Stimulator, Welwyn Garden City, UK) at 400 V, applied to the tibial nerve. A custom anode (Aluminium foil 10x6 cm and electrode paste) was applied to the anterior aspect of the thigh, proximal to the patella. A single Ag/AgCl electrode (Maxensor; Medimax Global, Australia), was located on the posterior side of the left knee within the popliteal fossa. To determine nerve location a rubber-insulated probe was manipulated within the popliteal fossa until the largest evoked resting H-wave was observed from the soleus EMG trace. To map the H-M curve, H-wave threshold was approximated by increasing stimulation intensity from 10 to 30 mA (1 mA increments). The start of the H-M curve was defined as three mA lower than the intensity to evoke ≥ 1 mV. To approximate M_max_, the stimulus intensity was increased by 10 mA increments until a plateau was achieved over three stimulation points, with the last point defined as M_max_. Thirty stimulation points were created on a logarithmic scale between these two approximations. Two stimulations (with 10 seconds rest between) were recorded for each of the thirty points.

### H-wave data processing

**Fig 3:**
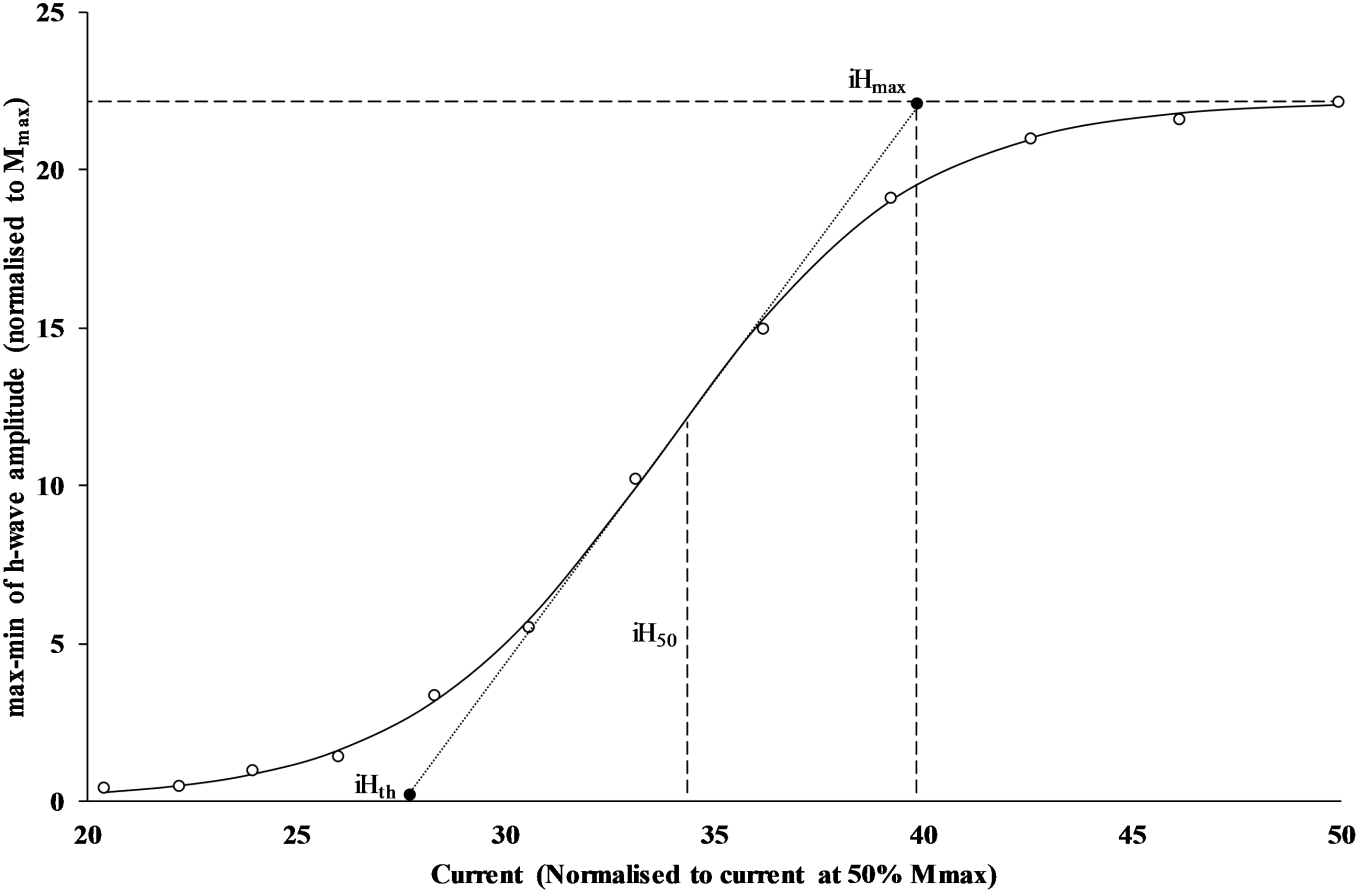
H-reflex parameters displayed on a representative example. The raw data is represented as open circles, with the sigmoid fit data represented along the solid line. The short dashed line represents the slope at 50% of H_max_. The lower filled circle represents iH_th_, and the upper filled circle represents iH_max_.

For H-wave processing, the ascending limb was defined as data from the initial stimulation point to H_max_. Data from the ascending limb was entered into a custom program coded in the statistical package R (R Foundation for Statistical Computing, Vienna, Austria). This program incorporated a three-parameter sigmoid function and used a general least squares model to find the best fit for the ascending limb H-wave data (Klimstra & Zehr, 2008). Recruitment curves with an R^2^ < 0.90 were omitted from analysis (Vila-Cha et al., 2012). The following parameters were acquired from the ascending limb: the slope at the midpoint (H_slp_), current at H-wave threshold (iH_th_), current at H-wave maximum amplitude (iH_max_) and current at 50% H_max_ (iH_50_). These parameters are visually represented in Fig 3. V-wave data was processed by normalising the value to the maximal motor response for that time point (M_max_).

### Statistical analyses

All statistical analyses were completed using IBM SPSS Statistics version 22 (SPSS Inc., Chicago, IL, USA), two-tailed statistical significance was accepted at P<0.05. The Kolmogorov-Smirnov test was applied to assess normality of distribution. The descriptive data are presented as means ± standard deviation (SD). All measurements were analysed by a two-way (condition x time) ANOVA for repeated measures (T1, T2 and T3). In the event of a significant F ratio, *post hoc* comparisons were made using a Bonferroni correction. Mean differences and standard deviation were reported in results tables. When significant changes over time or when differences between conditions were observed, data was reported as percentage change with the upper and lower 95% confidence intervals (CI) displayed in square brackets. The estimated effect size Cohen’s d (*d)* was reported for *post hoc* analysis.

## Results

### Hormonal analysis

**Fig 4:**
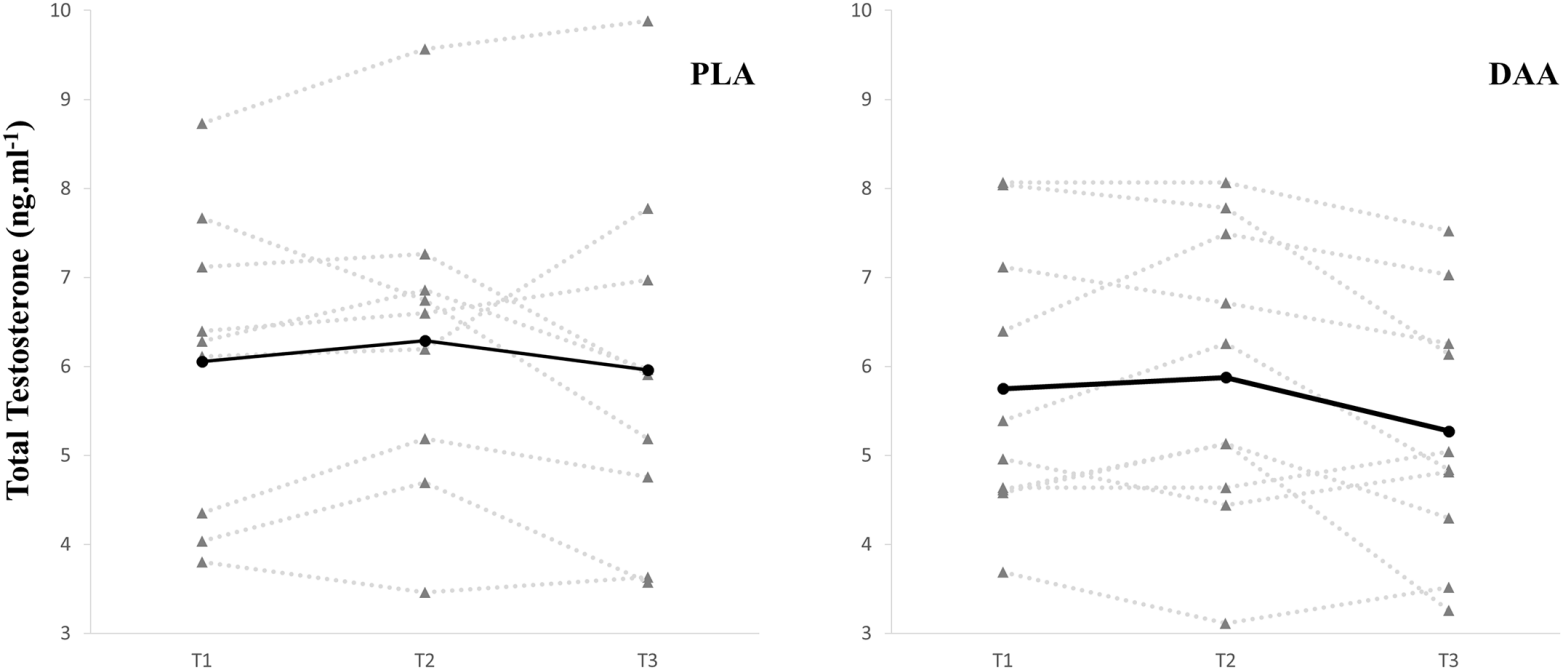
Individual response of total testosterone. Left graph depicts placebo data and right graph depicts the data from the d-aspartic group. Solid line depicts group mean.

**Fig 5:**
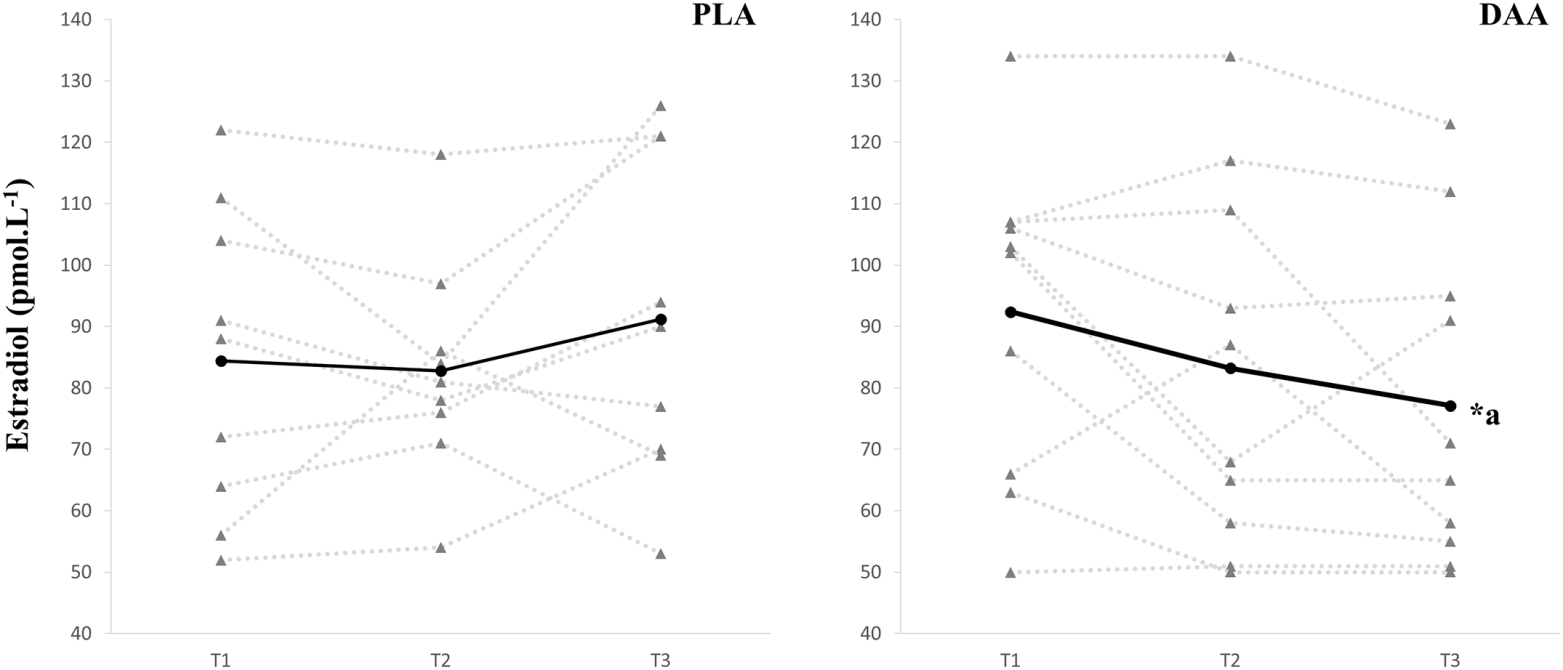
Individual response of estradiol. Left graph depicts placebo data and right graph depicts the data from the d-aspartic group. Solid line depicts group mean. *^a^ significant between group difference, as compared to T1 (p<0.01).

There was no main effect of time for the blood markers, FT (p = 0.661), SHBG (p = 0.180), ALB (p = 0.096) and a trend for a time effect for TT (p = 0.075, *d* = 0.18, see Fig 4, trend indicates an overall reduction in TT). Results from E_2_ revealed a significant group by time effect (p = 0.023) with *post hoc* analysis showing that the DAA group experienced a 16.2%, 95% CI [-27.0%, -5.5%] reduction in E_2_ from T1 to T3 (p = 0.009, *d* = 0.59, Fig 5). All other blood markers showed no significant group by time effect (TT, p = 0.614; FT, p = 0.543; SHBG, p = 0.829; ALB, p = 0.393; HDL, p = 0.301) (Table 3).

**Table 3:** Fasting hormones. Total testosterone (TT, ng/ml), free testosterone (FT, pmol/l), estradiol (E_2_, pmol/l), sex-hormone-binding-globulin (SHBG, nmol/l), albumin (ALB, g/l) levels, for placebo (PLA) and six grams per day of d-aspartic acid (DAA), at baseline (T1), six weeks midpoint (T2) and 12 weeks post testing (T3).

	PLA (n = 9)			DAA (n = 10)		
	T1	T2	T3	T1	T2	T3
TT	6.1 ± 1.5	6.3 ± 1.5	6.0 ± 1.9	5.7 ± 1.6	5.9 ± 1.6	5.3 ± 1.4
FT	431.9 ± 113.3	443.3 ± 121.2	444.4 ± 164.2	408.3 ± 90.3	418.7 ± 108.7	387.9 ± 95.1
E_2_	84.4 ± 25.0	82.8 ± 17.7	91.2 ± 26.5	92.4 ± 25.7	83.2 ± 29.5	77.1 ± 26.4*^a^
SHBG	36.2 ± 12.5	38.2 ± 14.5	34.6 ± 11.7	34.5 ± 15.3	34.6 ± 12.8	32.3 ± 10.2
ALB	45.4 ± 2.1	44.7 ± 3.1	44.6 ± 1.7	45.9 ± 2.5	46.1 ± 2.5	44.9 ± 1.9

Data are mean±SD.

*^a^ significant between-group difference, as compared to T1 (p<0.01).

### Isometric and dynamic muscle strength

**Fig 6:**
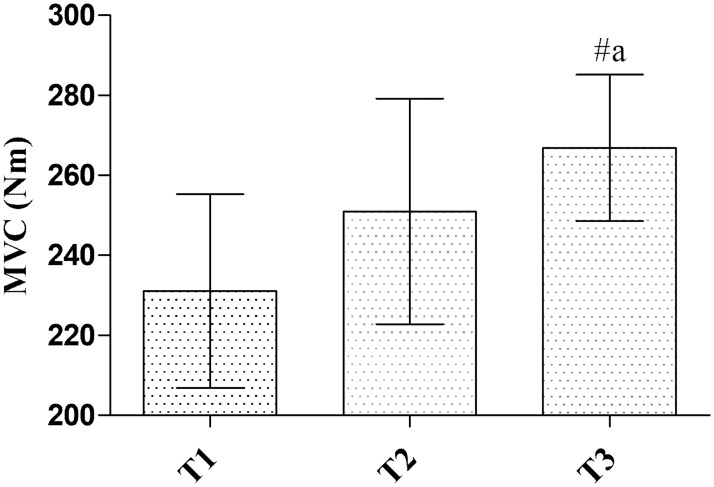
Maximal voluntary contraction results for isometric plantar flexor strength. Values at baseline (T1), six weeks midpoint (T2) and 12 weeks post testing (T3), n = 18. Data are mean, 95% CI. ^#a^ significantly different from T1, irrespective of group (p<0.01).

A main effect for time was observed for plantar flexor strength (p = 0.004, Table 4). *Post hoc* analysis revealed that isometric plantar flexor strength increased 17.2%, 95% CI [6.9%, 27.5%] from T1 to T3 (p = 0.008, *d* = 0.66, Fig 6). A significant main effect for time was observed in the 10RM seated calf raise (p<0.001, Table 4). *Post hoc* analysis revealed that dynamic calf strength was significantly increased by 50.3%, 95% CI [22.6%, 77.9%] from T1 to T2 (p<0.001, *d* = 1.24), 15.8%, 95% CI [9.2%, 22.3%] from T2 to T3 (p<0.001, *d* = 0.71) and 76.5%, 95% CI [37.7%, 115.3%] from T1 to T3 (p<0.001, *d* = 1.94).

**Table 4:** Maximal voluntary contractions and dynamic strength of the plantar flexors. Seated 90° calf raise MVC and seated 90° calf raise 10RM, for placebo (PLA) and six grams per day of d-aspartic acid (DAA), at baseline (T1), six weeks midpoint (T2) and 12 weeks post testing (T3).

	PLA (n = 8)			DAA (n = 10)		
	T1	T2	T3	T1	T2	T3
MVC	264.3 ± 52.0	292.8 ± 49.8	293.0 ± 40.8^#a^	243.6 ± 61.7	268.8 ± 78.8	287.6 ± 64.8^#a^
						
10 RM	33.9 ± 7.0	41.1 ± 6.5^#a^	47.8 ± 7.0^#^ ^@a^	31.3 ± 12.4	47.5 ± 10.5^#a^	53.7 ± 9.8^#a^ ^@a^
						

Data are mean±SD.

^#^ significantly different from T1, irrespective of group (p<0.05),

^#a^ (p<0.01).

^@a^ significantly different from T2, irrespective of group (p<0.01).

### α-motor neuron excitability and efferent drive

**Fig 7:**
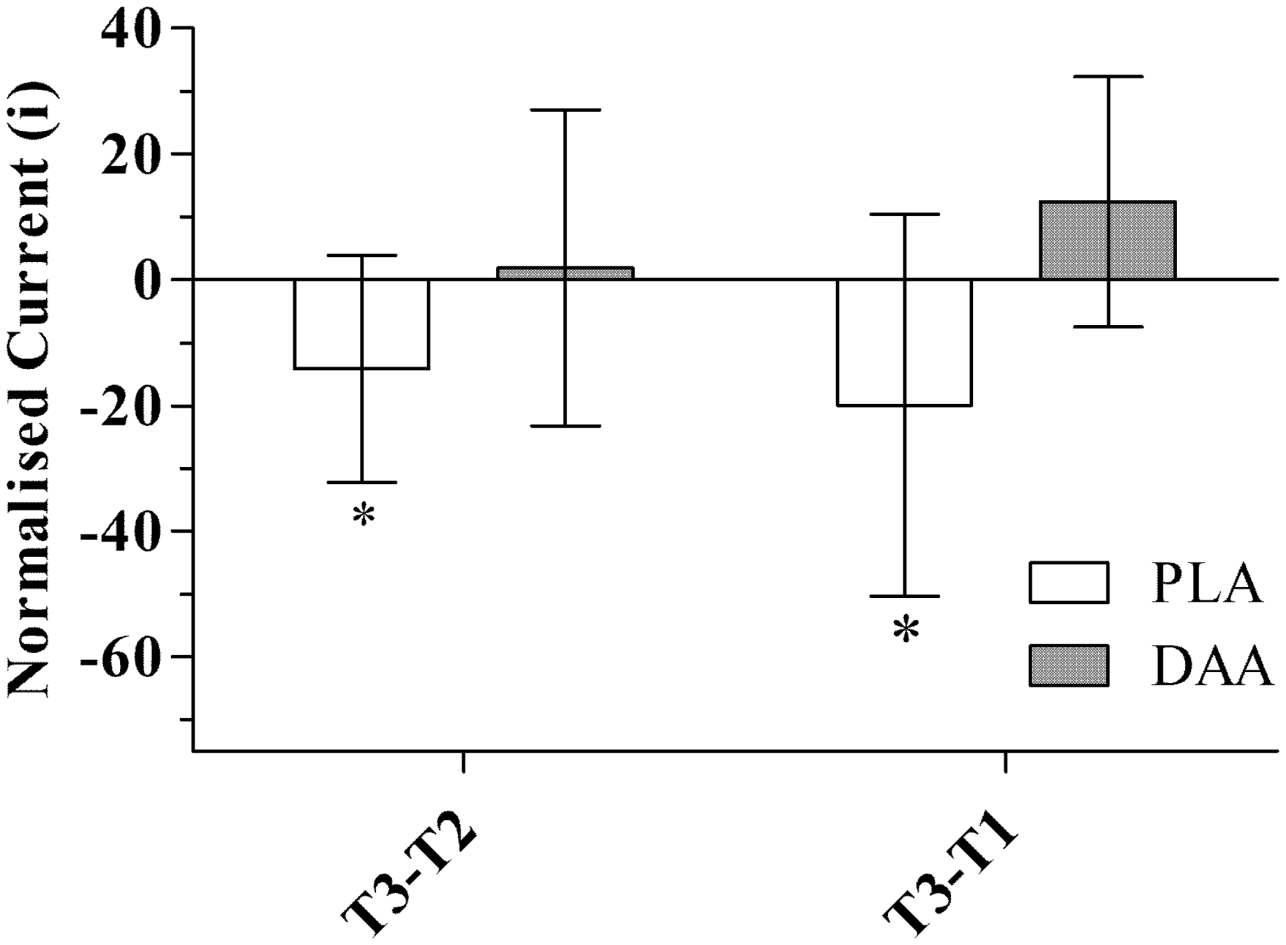
Absolute change score in gastrocnemius normalised iH_max_. Normalised to current at 50% M_max_, post testing minus midpoint testing (T3-T2) and post testing minus baseline testing (T3-T1). Data are mean, 95% CI. * significant difference between groups (p<0.05).

**Fig 8:**
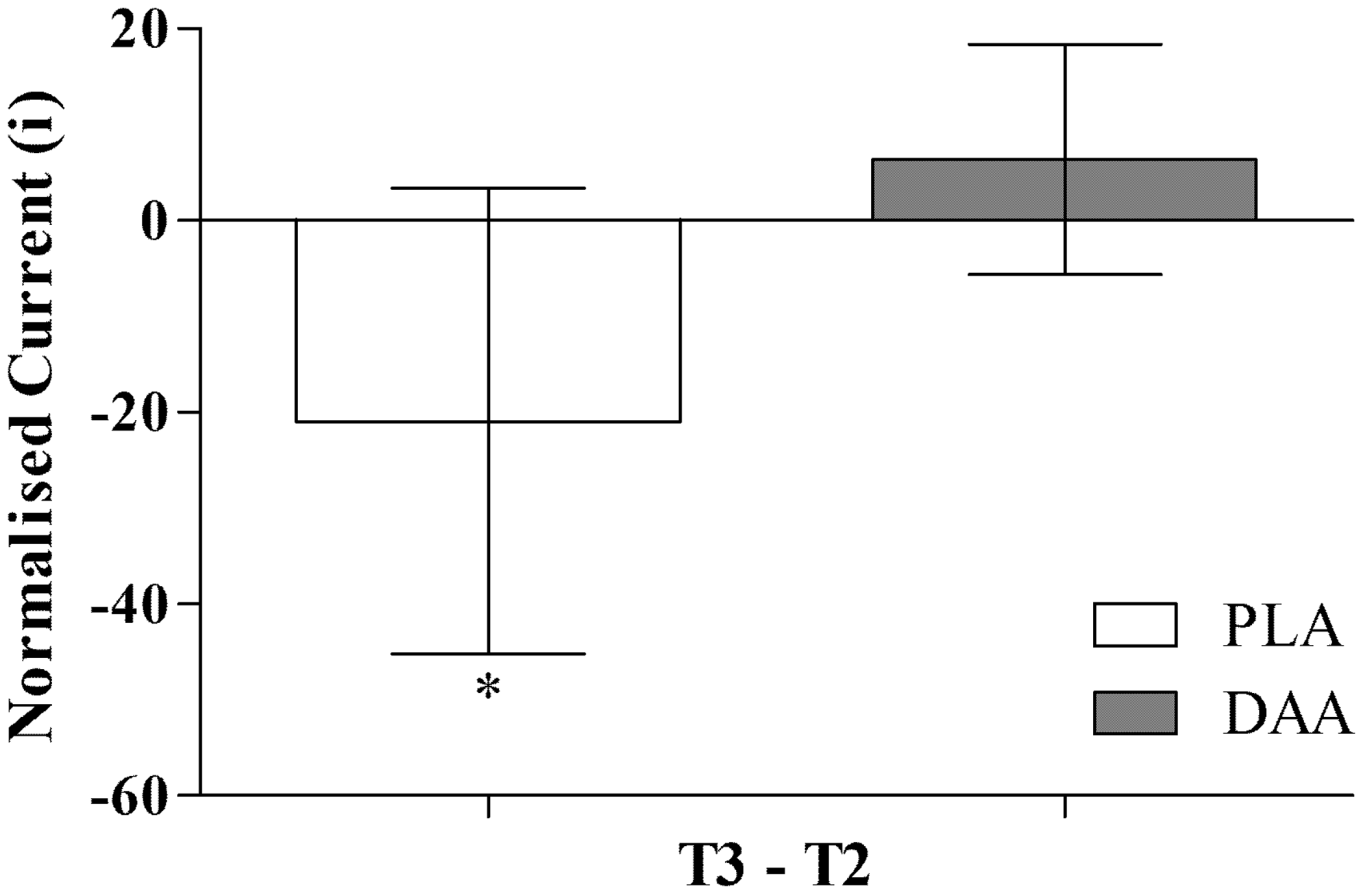
Absolute change score in gastrocnemius normalised iH_50_. Normalised to current at 50% Mmax, post testing minus midpoint testing (T3-T2). Data are mean, 95% CI. * significant difference between groups (p<0.05).

A main effect for time was observed for H_max_ (p = 0.023). *Post hoc* analysis revealed a decreasing trend between T1 and T3 (p = 0.101, *d* = 0.63). A significant group by time effect was observed in gastrocnemius iH_max_ (p = 0.031), and in iH_50_ (p = 0.033). *Post-hoc* analysis of iH_max_ showed a 13.8%, 95% CI [-36.0%, 8.4%] reduction from T2 to T3 in PLA (p = 0.038, *d* = 0.70; Fig 7). *Post-hoc* analysis for iH_50_ also demonstrated a 14.8%, 95% CI [-34.1%, 4.6%] reduction from T2 to T3 in PLA (p = 0.041, *d* = 0.68, Fig 8). No time effects (p>0.200) or group by time effects (p>0.100) were observed in any soleus H-reflex measure (Table 5). The V/M_max_ ratio had no main effect for time (V/M_max_, p = 0.417; V/M_max_, p = 0.587, soleus and gastrocnemius respectively) or group by time effect (V/M_max_, p = 0.568; V/M_max_, p = 0.496, respectively; Table 5).

**Table 5:** Plantar flexor neural variables. Maximal M-wave (M_max_, mV), V-wave to maximal M-wave ratio (V/M_max_, %), maximal H-wave (H_max_, % M_max_), slope at 50% H_max_ (H_slp_, mV/mA), current (i), all i variables normalised to current at 50% M_max_, current at H-wave threshold (iH_th_), current at 50% H_max_ (iH_50_), current at H_max_ (iH_max_), for placebo (PLA) and six grams per day of d-aspartic acid (DAA), at baseline (T1), six weeks midpoint (T2) and 12 weeks post testing (T3).

	PLA (n = 8)			DAA (n = 10)		
Soleus	T1	T2	T3	T1	T2	T3
M_max_	9.5 ± 3.5	8.0 ± 2.3	9.8 ± 4.4	9.8 ± 2.6	11.3 ± 5.4	8.9 ± 4.0
V/M_max_	30.46 ± 27.75	34.08 ± 29.28	26.49 ± 18.60	24.89 ± 9.33	30.37 ± 17.62	28.92 ± 20.29
H_max_	44.1 ± 18.3	47.4 ± 18.2	47.6 ± 15.4	45.3 ± 12.4	46.3 ± 17.4	39.8 ± 12.5
H_slp_	2.1 ± 1.4	2.6 ± 2.3	2.8 ± 1.7	2.1 ± 1.3	2.9 ± 2.3	2.3 ± 1.7
iH_th_	59.0 ± 27.0	50.8 ± 23.0	40.3 ± 8.8	47.8 ± 19.1	44.5 ± 17.1	49.8 ± 21.0
iH_50_	70.8 ± 29.5	62.4 ± 25.6	51.0 ± 9.5	60.8 ± 23.5	54.8 ± 20.1	62.5 ± 20.6
iH_max_	82.7 ± 32.3	74.1 ± 29.4	61.8 ± 12.5	73.8 ± 28.3	65.0 ± 23.7	75.2 ± 22.2
Gastrocnemius	T1	T2	T3	T1	T2	T3
M_max_	7.7 ± 3.0	9.0 ± 3.1	8.9 ± 3.5	9.7 ± 2.8	10.1 ± 3.5	10.7 ± 2.9
V/M_max_	26.50 ± 28.41	30.87 ± 28.76	24.50 ± 18.54	22.20 ± 9.91	25.30 ± 10.41	27.40 ± 30.24
H_max_	49.3 ± 22.3	34.9 ± 18.0	36.5 ± 20.4	41.7 ± 21.5	38.4 ± 18.6	31.4 ± 13.5
H_slp_	2.2 ± 1.3	1.9 ± 1.6	2.5 ± 2.0	2.1 ± 1.7	2.5 ± 1.7	1.6 ± 1.2
iH_th_	46.0 ± 18.7	54.6 ± 31.8	40.0 ± 8.1	47.5 ± 19.4	46.5 ± 19.3	44.6 ± 15.0
iH_50_	59.7 ± 20.2	66.9 ± 35.0	49.6 ± 7.3^ϕ^	60.9 ± 23.1	54.1 ± 20.3	61.5 ± 22.4
iH_max_	73.42 ± 23.5	79.2 ± 39.0	59.3 ± 9.1*^ϕ^	74.3 ± 28.1	63.8 ± 25.0	76.2 ± 29.0

Data are mean ± SD.

* significant between-group differences, as compared to T1 (p<0.05).

^**ϕ**^ significant between-group differences, as compared to T2 (p<0.05).

### Muscle hypertrophy and architecture

A significant main effect for time was observed in body mass (p = 0.005, Table 6). *Post hoc* analysis revealed a 1.5%, 95% CI [0.2%, 2.4%] increase in body mass from T1 to T3 (p = 0.045, *d* = 0.12). A significant main effect for time was observed for CSA in the muscles, VL (p<0.001), VI (p = 0.002), RF (p = 0.034) and VM (p<0.001). *Post-hoc* analysis of CSA revealed that VL was increased 6.5%, 95% CI [2.3%, 10.8%] from T1 at T2 (p = 0.020, *d* = 0.33) and 10.1%, 95% CI [5.4%, 14.8%] at T3 (p<0.001, *d* = 0.48). VM was increased 7.8%, 95% CI [4.3%, 11.3%] from T1 at T2 (p<0.001, *d* = 0.21) and 11.4%, 95% CI [6.4%, 16.4%] at T3 (p<0.001; *d* = 0.41). VI was increased 6.6%, 95% CI [2.3%, 10.9%] from T1 to T3 (p = 0.008 *d* = 0.41). RF demonstrated an increasing trend between T1 and T3 (p = 0.091, *d* = 0.41, Table 6).

**Table 6:** Body mass and ultrasound hypertrophy data. Vastus lateralis (VL), vastus intermedialis (VI), rectus femoris (RF), vastus medialis (VM), soleus (SOL), gastrocnemius (GAS). Percentage indicates the distance from distal to proximal with quadriceps images referenced from the centre knee joint to the ASIS and calf images referenced from lateral malleolus to the fibular head.

	Placebo (n = 9)			6 g/d (n = 10)		
	T1	T2	T3	T1	T2	T3
Body Mass	82.5 ± 9.0	83.2 ± 7.9	83.8 ± 8.0^#^	80.5 ± 10.2	81.0 ± 9.8	81.4 ± 9.9^#^
Quadriceps						
CSA (cm^2^)						
VL	22.4 ± 5.3	24.1 ± 4.9^#^	24.8 ± 4.7^#b^	21.1 ± 4.4	21.9 ± 3.6^#^	22.7 ± 4.1^#b^
VI	23.7 ± 4.1	24.4 ± 3.9	25.3 ± 3.6^#b^	26.6 ± 3.7	27.3 ± 3.9	27.9 ± 3.8^#b^
RF	4.1 ± 1.4	4.3 ± 0.8	4.6 ± 1.0	4.7 ± 1.2	5.1 ± 1.1	5.2 ± 1.2
VM	18.7 ± 4.1	19.6 ± 4.3^#b^	20.4 ± 4.1^#b^	18.3 ± 4.6	20.1 ± 4.8^#b^	20.5 ± 4.7^#b^
Sagittal (mm)						
VL_33%_	27.5 ± 3.7	28.5 ± 3.1	28.3 ± 4.0	26.4 ± 4.1	27.1 ± 4.9	27.7 ± 4.8
VI_33%_	17.2 ± 3.4	18.9 ± 2.7	18.4 ± 3.3	19.1 ± 4.0	19.5 ± 4.2	19.8 ± 3.4
VL_50%_	25.8 ± 3.6	26.6 ± 3.5	26.6 ± 4.6	26.0 ± 4.7	27.1 ± 5.5	27.1 ± 4.7
VI_50%_	17.2 ± 3.4	18.9 ± 2.7	18.4 ± 3.3	20.1 ± 4.6	20.6 ± 4.7	20.5 ± 4.6
						
VL_Angle_ (°)	14.4 ± 2.6	15.6 ± 3.0	16.4 ± 3.7	15.9 ± 3.4	17.5 ± 4.6	16.6 ± 3.2
Calf						
Sagittal (mm)						
SOL_75%_	14.0 ± 2.5	13.3 ± 2.6	14.0 ± 2.3	14.1 ± 2.1	13.6 ± 1.5	14.2 ± 1.4
SOL_67%_	14.3 ± 2.3	14.7 ± 2.2	14.9 ± 2.2	14.1 ± 2.2	14.3 ± 2.2	14.9 ± 2.3
SOL_50%_	14.8 ± 2.2	17.4 ± 5.7	16.2 ± 3.0	14.2 ± 3.5	15.5 ± 2.6	16.1 ± 2.2
						
GAS_75%_	8.6 ± 3.0	9.4 ± 3.2^#a^	10.4 ± 2.4^#^	9.4 ± 1.6	10.1 ± 1.5^#a^	10.1 ± 1.5^#^
GAS_67%_	8.5 ± 3.0	9.8 ± 3.0	10.6 ± 2.9^#^	9.4 ± 2.5	9.7 ± 3.0	10.1 ± 2.5^#^
GAS_50%_	3.8 ± 2.0	4.2 ± 1.8	4.4 ± 1.3	4.5 ± 3.0	3.2 ± 1.0	3.8 ± 0.9

Data are mean±SD.

^#^ significantly different from T1, irrespective of group (p<0.05),

^#a^ (p<0.01),

^#b^ (p<0.001)

For the quadriceps sagittal images (Table 6), a significant main effect for time was observed in VI_33%_ (p = 0.047) and VL_Angle_ (p = 0.040; *d* = 0.41). *Post-hoc* analysis revealed no significant change from T1 to T3, in VI_33%_ (p = 0.135; *d* = 0.26) and VL_A_ (p = 0.106; *d* = 0.41). No significant main effects for time were observed in soleus at SOL_75%_ (p = 0.217), SOL_67%_ (p = 0.141), with an increasing trend observed in SOL_50%_ (p = 0.063, d = 0.49). Significant main effects for time were observed in the gastrocnemius muscle at GAS_75%_ (p<0.001) and GAS_67%_ (p = 0.004) but not GAS_50%_ (p = 0.468). *Post-hoc* analysis revealed that GAS_75%_ increased 9.7%, 95% CI [4.3%, 15.0%] from T1 to T2 (p = 0.002, *d* = 0.32) and 18.6%, 95% CI [4.8%, 32.3%] from T1 to T3 (p = 0.011, *d* = 0.59). GAS_67%_ increased 20.6%, 95% CI [3.4%, 37.9%] from T1 to T3 (p = 0.012, *d* = 0.51; Table 6).

## Discussion

The short-term supplementation (14 days) has been shown to reduce testosterone levels in resistance-trained men [11]. Thus the primary objective of this study was to evaluate the effect of DAA supplementation on basal testosterone over a three month resistance training period. Based on our previous results, we hypothesised that DAA would reduce basal testosterone levels. The novel findings of this study were that 1) DAA did not increase or decrease testosterone levels in resistance-trained men, 2) DAA supplementation reduced levels of estradiol, 3) equivalent strength and hypertrophy gains were observed for both the placebo and DAA groups, and 4) the placebo group experienced a reduction in all gastrocnemius H-reflex parameters pertaining to current intensity.

### Effects of DAA on hormones, strength and hypertrophy

Three months of DAA supplementation did not change basal testosterone levels in resistance-trained men. The lack of change observed in the present study suggests that the previously observed reduction in testosterone after two weeks supplementation may be transitory [11]. Previous research in resistance-trained men has observed increases in d-aspartate oxidase [12], which oxidises DAA. It is plausible that d-aspartate oxidase production is linked with total testosterone levels, and regulates abnormal levels of DAA in the bloodstream. This could explain why increased testosterone from DAA supplementation has been observed when levels of testosterone are low-normal [6], in comparison to the lack of change observed when testosterone levels are normal-high [11, 12]. The results of this study clearly demonstrate that DAA is an ineffective supplement for improving basal testosterone levels in resistance-trained men.

The supplementation of DAA caused a marked reduction in estradiol levels. Mechanistically, estradiol was potentially reduced via disruption of the testosterone-estradiol aromatase pathway [29]. In the animal model, both positive [30] and negative [31] relationships have been observed between gonadal DAA and estradiol. Additionally, *in vitro* research has observed both increased [30, 32] and decreased [31] estradiol levels with DAA supplementation. With this conflicting data, the effects of DAA on estradiol in the animal model is unclear. In resistance-trained men estradiol remains unchanged over two [11] and four weeks [12] from a resistance training and supplement protocol. Despite an observed reduction of estradiol in the present study, the training outcomes clearly demonstrate similar improvements in hypertrophy and strength. This is further evidence that basal hormonal fluctuations within normal ranges are not key explanatory mechanisms of training outcomes, rather it is likely intrinsic mechanisms that explain training improvements and that hormones play a permissive role in training adaptions. Intrinsic mechanisms pertaining to hypertrophy include the activation of various signalling pathways (Akt/mTOR, PA/mTOR, mechanoreceptor), phosphorylation of intramuscular signalling proteins, regulation of messenger RNA (translation initiation), increases in AR content, satellite cell activity and expression of muscle-specific microRNA [33, 34]. For strength it appears that neural adaptation mechanisms such as earlier recruitment of type II motor units, as demonstrated by a reduction in recruitment threshold distributions; increased observation of ‘true’ doublet spikes; or increased maximal firing frequency of motor units during voluntary ballistic contractions shown by a reduction of inter-spike intervals; increase in efferent neural drive to the muscle: and an increase in the firing frequency of the motor units [15, 16, 35] are driving improvments.

### Neural adaptation

This is the first study to examine changes in the H-reflex pathway following a period of resistance exercise in trained men. These results demonstrate for the first time improvements in responsiveness at the level of the alpha motoneuron, similar to that which is observed in novice populations [24, 36, 37]. Mechanistically, this could be a result of improved excitability of the alpha motoneuron, improved excitability of the Ia afferent loop, or a decrease in presynaptic inhibition at the level of the interneuron [38].

Improved excitability of the H-reflex pathway in the placebo group was blunted by DAA supplementation. It is plausible since DAA appears to fit the role of a neurotransmitter [21], that DAA is inhibiting the capacity of the neurotransmitter system to adapt over time to the resistance exercise stimulus. DAA can enter the neuron via L-glutamate transporters [39, 40]. However, the transport of DAA could be saturating the L-glutamate transporters. This, in turn, might inhibit a more potent neurotransmitter, such as glutamate [41] and ultimately result in blunted neural adaption. As both groups equally improved plantar flexor isometric and dynamic strength (10RM values), the contribution of between-group differences observed in excitability of the spinal pathway appears negligible. The H-reflex utilises the same pathway as the myotatic reflex [42], which is important reflex in the context of power development because it activates alpha motoneuron activity in a rapidly stretched muscle [38]. A potential limitation of this study is the lack of testing for dynamic power (e.g. plyometric testing), as this would help clarify the relevance of the improved H-reflex excitability from the perspective of functional power.

The present study showed that V/M_max_ ratio in the plantar flexors did not improve over the course of the training study, suggesting that neither DAA supplementation nor training affected supraspinal drive to the plantar flexors in this population. Research in novice populations has demonstrated increases in V/M_max_ ratio with resistance training [16, 24, 43, 44], however resistance-trained individuals (e.g. weightlifters) demonstrate significantly larger V-wave responses in comparison to inexperienced trainers [45]. Data on endurance training has failed to show any improvements in V-wave measures, suggesting that the improvement of neural drive is an adaptation exclusive to resistance training [24]. The results of the current study present novel evidence, demonstrating that resistance-trained individuals do not improve supraspinal drive to the plantar flexors with three months of resistance training. This could suggest that there is a ceiling for adaptation of supraspinal drive and that further neural improvements are exclusive to changes of excitability within the spinal cord. The data from the present study exhibited high variability between subject’s V-waves, which might suggest an issue with the sensitivity of the method that was used. Additionally, the reliance of V-waves to measure a change in supraspinal drive is a limitation of this study. The inclusion of additional measures of corticospinal function from the use of transcranial stimulation techniques may have provided clearer insight into potential supraspinal changes.

### Practical implications and future research direction

DAA is currently purported as a testosterone boosting supplement. The results of this study not only suggest otherwise but also add to the body of evidence that changes in basal hormonal levels within the normal physiological range play a minor role with respect to training outcomes. The long-term effects of DAA did not provide any benefit in relation to strength or hypertrophy in a resistance-trained population. Additionally, DAA appears to be blunting neural adaptation that was evident in the placebo group. As such, the results of this study strongly suggest that DAA is not an ideal supplement for resistance-trained men and cannot be recommended for long-term use with resistance training.

The effects of DAA have yet to be explored in either a female population, where mechanisms of DAA and testosterone manipulation could differ. Additionally, an elderly population, with decreased testosterone levels, may benefit from DAA supplementation combined with resistance training, as some research has demonstrated that DAA can improve testosterone levels in untrained men. The mechanisms driving power adaptations in untrained individuals are well researched, however, in a resistance-trained population the data is lacking. The link between neural plasticity and changes in functional power from resistance training, from a resistance-trained context could also be explored. Additionally, future studies could further test the theory that resistance-trained individuals experience a ceiling effect with supraspinal neural adaptations, or if they have the ability to continue to improve supraspinal drive.

## Supporting information

S1 File Consort checklist document. (DOC)

S2 File NEAF—Original study protocol approved by ethics in 2013. (PDF)

S3 File PIS—Study protocol approved by ethics in 2014. (DOCX)
