# Supplementary material for: The effects of d-aspartic acid supplementation in resistance-trained men over a three month training period: A randomised controlled trial
Source: PLoS One. 2017 Aug 25;12(8):e0182630. doi: 10.1371/journal.pone.0182630 (PMC5571970; doi:10.1371/journal.pone.0182630)
Supplement: S2 File — (PDF) [file pone.0182630.s002.pdf]

National Ethics Application Form

Version 2008 - V2.0

Proposal title: **The effect of D-Aspartic Acid on adaptation from a 24 week resistance training program**

For submission to:
University of Western Sydney Human Research Ethics Committee (EC00314)

Name: **Dr Paul Marshall**

Address: **Building 20 G Room 35**
School of Science and Health
UWS Campbelltown Campus
Locked Bag 1797
Penrith South DC NSW 1797

Contact: (Bus) **0246203915**
(AH) **-**
(Mob) **-**
(Fax) **0234567890**

Proposal status: **Complete**

Proposal description:

Aims:
Our aim is to implement two studies; the first will measure the effect that d-aspartic acid supplementation has on basal testosterone levels in advanced trainers, whilst they are undergoing standardised training. The second study will explore effects of d-aspartic acid supplementation on strength, hypertrophy and neural adaptation with concurrent resistance training over a period of 6 months.
Primary Objectives

Study 1

• To measure change in basal testosterone levels from three different doses of DAA (0g, 3g, 6g).
• To examine the effect of the recommended (3g) dose compared to a double-dose (6g) protocol.

Study 2

• To determine the relationship between d-aspartic acid and strength after 24 weeks of resistance training
• To determine the relationship between testosterone change and neural adaptation after 24 weeks of resistance training
• To determine the relationship between testosterone change and hypertrophy after 24 weeks of resistance training

Proposal research design:

The first study (acute) will be a double blinded randomised controlled trial that will explore two dosing strategies of DAA. The cohort will comprise of men experienced in resistance training who are currently training. This study will be conducted over 4-weeks, with an initial 2-week

standardisation period.At the end of the two week standardisation period baseline blood draws will be taken. Then participants will be allocated to one of three groups D6, D3, D0 which will be 6 grams, 3 grams and 0 grams of DAA respectively. Participants will consume their relevant dose daily for 12 days

The second study will be a double blinded randomised controlled trial that will compare the outcomes of the participants assigned to the experimental treatment groups with those assigned to the placebo control group. The cohort will comprise of healthy participants experienced in resistance training. Participants will be randomised to their group, either 3g D-Asp (D3), 6g D-Asp (D6) or placebo (P) then begin their daily allocated dose and resistance training program for 12 weeks. After training for 12 weeks both groups will have a one week break (de-load week) to help prevent overtraining. They will then continue training for another 12 weeks. 5 blood draws will be taken in total. These will be conducted at baseline, 12 days, 30 days, 90 days and 180 days.

Previously submitted to:

Administrative Section

1. TITLE AND SUMMARY OF PROJECT

1.1. Title

1.1.1 What is the formal title of this research proposal?

The effect of D-Aspartic Acid on adaptation from a 24 week resistance training program

1.1.2 What is the short title / acronym of this research proposal (if applicable)?

Effects of DAA during resistance training

1.2. Description of the project in plain language

1.2.1 Give a concise and simple description (not more than 400 words), in plain language, of the aims of this project, the proposal research design and the methods to be used to achieve those aims.

Aims:
Our aim is to implement two studies; the first will measure the effect that d-aspartic acid supplementation has on basal testosterone levels in advanced trainers, whilst they are undergoing standardised training. The second study will explore effects of d-aspartic acid supplementation on strength, hypertrophy and neural adaptation with concurrent resistance training over a period of 6 months.
Primary Objectives

Study 1

- To measure change in basal testosterone levels from three different doses of DAA (0g, 3g, 6g).
- To examine the effect of the recommended (3g) dose compared to a double-dose (6g) protocol.

Study 2

- To determine the relationship between d-aspartic acid and strength after 24 weeks of resistance training
- To determine the relationship between testosterone change and neural adaptation after 24 weeks of resistance training
- To determine the relationship between testosterone change and hypertrophy after 24 weeks of resistance training

Proposal research design:

The first study (acute) will be a double blinded randomised controlled trial that will explore two dosing strategies of DAA. The cohort will comprise of men experienced in resistance training who are currently training. This study will be conducted over 4-weeks, with an initial 2-week standardisation period.At the end of the two week standardisation period baseline blood draws will be taken. Then participants will be allocated to one of three groups D6, D3, D0 which will be 6 grams, 3 grams and 0 grams of DAA respectively. Participants will consume their relevant dose daily for 12 days

The second study will be a double blinded randomised controlled trial that will compare the outcomes of the participants assigned to the experimental treatment groups with those assigned to the placebo control group. The cohort will comprise of healthy participants experienced in resistance training. Participants will be randomised to their group, either 3g D-Asp (D3), 6g D-Asp (D6) or placebo (P) then begin their daily allocated dose and resistance training program for 12 weeks. After training for 12 weeks both groups will have a one week break (de-load week) to help prevent overtraining. They will then continue training for another 12 weeks. 5 blood draws will be taken in total. These will be conducted at baseline, 12 days, 30 days, 90 days and 180 days.

2. RESEARCHERS / INVESTIGATORS

2.1. Chief researcher(s) / investigator(s)

**2.1.0 How many chief researchers / investigators are there? This question only applies to multi-centre research. If your research is not multi-centre, please enter 0 (zero) here. See Guidance Text (G) for the definition of a Chief Researcher.**	0

2.2. Principal researcher(s) / investigator(s)

**2.2.0 How many principal researchers / investigators are there?**	3

2.2.1. Principal researcher / investigator 1

2.2.1. Name and contact details

Name:	Mr Geoffrey Melville		
Address:	Building 20 G Room 35<br>School of Science and Health<br>UWS Campbelltown Campus<br>Locked Bag 1797<br>Penrith South DC NSW 1797<br>Australia		
Organisation:	University of Western Sydney		
Area:	School of Science and Health		
Position:	PhD Candidate		
Contact	(Bus) 0426263496<br>(Mob) -	(AH) 0426263496<br>(Fax) 0246203020	
Email:	16358238@student.uws.edu.au		

2.2.2... Summary of qualifications and relevant expertise NS 4.8.7 NS 4.8.15
Bachelor of Applied Science (Sport & Exercise Science) (Hons)

2.2.2... Please declare any general competing interests
There are no general competing interests

2.2.2... Name the site(s) for which this principal researcher / investigator is responsible.
The research will be conducted at the University of Western Sydney (Campbelltown Campus).

2.2.3 Describe the role of the principal researcher / investigator in this project.
The roles of Principle Investigator 1 include:
1. Completion of the ethics application
2. Development and implementation of research methodology
3. Recruitment of participants
4. Testing of participants (i.e. data collection)
5. Statistical analyses
6. Providing written feedback to the participants
7. Writing of the thesis
8. Preparation of manuscripts and abstracts for publication and presentation at international congresses

**2.2.4 Is the principal researcher / investigator a student?**	Yes

2.2.4...What is the educational organisation, faculty and degree course of the student?

**Organisation**	University of Western Sydney
**Faculty**	School of Science and Health
**Degree course**	Doctor of Philosophy

**2.2.4... Is this research project part of the assessment of the student?**	Yes
**2.2.4... Is the student's involvement in this project elective or compulsory?**	Compulsory

2.2.4... What training or experience does the student have in the relevant research methodology?
A training course in phlebotomy has been completed (15/10/2012): Adept Training - Perform Blood Collection (HLTPA306C) at Liverpool NSW.

Development and practise of measuring muscle hypertrophy using B-Mode ultrasonography.

The student has previously completed unit 400872: Research Design and Methodology in 2010 during honours year.

2.2.4... What training has the student received in the ethics of research?

The student has read the material provided via the University website overviewing ethics in research. An ethics class has been provided through the subject: Research Design and Methodology (UWS unit code: 400872), which was completed in 2010. The student has also been provided with specific research ethics guidance by Dr. Paul Marshall and Dr Jason Siegler.

2.2.4... Describe the supervision to be provided to the student. NS 4.8.8

The student will receive direct academic supervision from Dr. Paul Marshall who is a lecturer in the School of Science and Health at the University of Western Sydney. This supervision will include fortnightly one-on-one meetings with the student to discuss project progress, and direct methodology supervision to ensure the appropriate skill sets are learned and conducted in accordance with approved safety standards.

2.2.4... How many supervisors does the student have? 2

2.2.4...Supervisor 1

2.2.4...Provide the name, qualifications, and expertise, relevant to this research, of the students' supervisor

**Title**	Dr
**First Name**	Paul
**Surname**	Marshall
**Summary of qualifications and relevant expertise**	PhD Exercise Physiology<br>PG Dip Sci Exercise Physiology<br>BSc Sport and Exercise Science<br>BCom Management and Employment Relations<br><br>Over 30 peer reviewed publications specializing in exercise physiology<br><br>16 years clinical experience of exercise prescription<br><br>Supervision of approximately 40 Hons and PG Dip Sci<br><br>Research Students

2.2.4...Supervisor 2

2.2.4...Provide the name, qualifications, and expertise, relevant to this research, of the students' supervisor

**Title**	Dr
**First Name**	Jason
**Surname**	Siegler
**Summary of qualifications and relevant expertise**	Dr Jason Siegler has extensive experience in study development, subject recruitment, data collection and analysis. Dr. Siegler has conducted and published independent research on many aspects of physiology, sport nutrition and performance. He has a Ph.D. in Exercise Physiology and has extensive experience in exercise testing of athletes and other populations. He is a trained phlebotomist and holds certifications in Advanced Cardiac Life Support (ACLS) and Basic Life Support (BLS).

2.2.1. Principal researcher / investigator 2

2.2.1. Name and contact details

Name:	Dr Paul Marshall
Address:	Building 20 G Room 35<br>School of Science and Health<br>UWS Campbelltown Campus<br>Locked Bag 1797

Penrith South DC NSW 1797

Organisation: University of Western Sydney

Area: School of Science and Health

Position: Senior Lecturer

Contact (Bus) 0246203915 (AH) -
(Mob) - (Fax) 0234567890

Email: p.marshall@uws.edu.au

2.2.2... Summary of qualifications and relevant expertise NS 4.8.7 NS 4.8.15

PhD Exercise Physiology
PG Dip Sci Exercise Physiology
BSc Sport and Exercise Science
BCom Management and Employment Relations

Over 30 peer reviewed publications specializing in exercise physiology

16 years clinical experience of exercise prescription

Supervision of approximately 40 Hons and PG Dip Sci Research Students

2.2.2... Please declare any general competing interests

There are no competing interests

2.2.2... Name the site(s) for which this principal researcher / investigator is responsible.

University of Western Sydney, Campbelltown

2.2.3 Describe the role of the principal researcher / investigator in this project.

PhD supervisor

2.2.4 Is the principal researcher / investigator a student? No

2.2.1. Principal researcher / investigator 3

2.2.1. Name and contact details

Name: Dr Jason Siegler

Address: Building 20 G Room 35
School of Science and Health
University of Western Sydney
Locked Bag 1797
Penrith South DC NSW 1797

Organisation: University of Western Sydney

Area: School of Science and Health

Position: Director of Academic Program, Sport & Exercise

Contact (Bus) 02 4620 3381 (AH) -
(Mob) - (Fax) 02 4620 3792

Email: j.siegler@uws.edu.au

2.2.2... Summary of qualifications and relevant expertise NS 4.8.7 NS 4.8.15

Dr Jason Siegler has extensive experience in study development, subject recruitment, data collection and analysis. Dr. Siegler has conducted and published independent research on many aspects of physiology, sport nutrition and performance. He has a Ph.D. in Exercise Physiology and has extensive experience in exercise testing of athletes and other populations. He is a trained phlebotomist and holds certifications in Advanced Cardiac Life Support (ACLS) and Basic Life Support (BLS).

2.2.2... Please declare any general competing interests

There are no competing interests

2.2.2... Name the site(s) for which this principal researcher / investigator is responsible.

University of Western Sydney (Campbelltown Campus)

2.2.3 Describe the role of the principal researcher / investigator in this project.

PhD supervisor

2.2.4 Is the principal researcher / investigator a student? No

2.3. Associate researcher(s) / investigator(s)

2.3.1 How many known associate researchers are there? (You will be asked to give contact details for these associate researchers / investigators at question 2.3.1.1) 0

2.3.2 Do you intend to employ other associate researchers / investigators? No

2.4. Contact

Provide the following information for the person making this application to the HREC.

2.4.1. Name and contact details

Name:	Dr Paul Marshall
Address:	Building 20 G Room 35<br>School of Science and Health<br>UWS Campbelltown Campus<br>Locked Bag 1797<br>Penrith South DC NSW 1797
Organisation:	University of Western Sydney
Area:	School of Science and Health
Position:	Senior Lecturer
Contact	(Bus) 0246203915 (AH) -<br>(Mob) - (Fax) 0234567890
Email:	p.marshall@uws.edu.au

2.5. Other personnel relevant to the research project

2.5.1 How many known other people will play a specified role in the conduct of this research project? 2

2.5.1... Describe the role, and expertise where relevant (e.g. counsellor), of these other personnel.

Two Honours students will be involved in assisting with the implementation of the exercise intervention.

Details to be advised to the Human Ethics Officer as an amendment as they become available.

2.5.2 Is it intended that other people, not yet known, will play a specified role in the conduct of this research project? No

2.6. Certification of researchers / investigators

2.6.1 Are there any relevant certification, accreditation or credentialing requirements relevant to the conduct of this research? No

2.7. Training of researchers / investigators

2.7.1 Do the researchers / investigators or others involved in any aspect of this research project require any additional training in order to undertake this research? Yes

2.7.1... What is this training?

The primary researcher has undertaken a phlebotomy course.

2.7.1... How and by whom will the training be provided?

The course was provided by Adept training over two days encompassing both written and practical with assessments in both fields.

2.7.1... How will the outcome of the training be evaluated?

The training will be evaluated via successful completion of the two day course and successful completion of the practical and written assessments.

This course has been successfully completed by Geoffrey Melville.

3. RESOURCES

3.1. Project Funding / Support

3.1.1. Indicate how the project will be funded

3.1.1... Type of funding.
[Please note that all fields in any selected funding detail column (with the exception of the code) will need to be completed.]

	Internal Competitive Grant	Sponsor
Name of Grant / Sponsor	HDR Candidate Support Funding	Bulk Nutrients
Amount of funding	14000	15-20 kgs
Confirmed / Sought	Sought	Sought
Detail in kind support	Higher Degree Research, internal funding support $7000 per year	Provision of supplement: D-Aspartic Acid
Indicate the extent to which the scope of this HREC application and grant are aligned	The HDR funding is directly related to this HREC application.	The provision of this supplement is directly related to this HREC application.

3.1.1... How will you manage a funding shortfall (if any)?
N/A

3.1.2 Will the project be supported in other ways eg. in-kind support/equipment by an external party eg. sponsor Yes

3.1.2... Describe the support and indicate the provider.
Bulk Nutrients:

Supplements required for the study, D-Aspartic Acid

3.1.3... Is this a study where capitation payments are to be made, and will participants be made aware of these payments to clinicians or researchers / investigators? NS 3.3.18b
No

3.2. Duality of Interest

3.2.1 Describe any commercialisation or intellectual property implications of the funding/support arrangement.
The company Bulk Nutrients will use the outcomes of this study to promote their d-aspartic product, if the outcomes of the research prove favourable. Bulk nutrients has no proprietary rights to the supplement and thus the outcome(s) of the research is available to all suppliers of the supplement, once the research has been published. There will be no direct commercial benefit from the outcomes of this study.

3.2.2 Does the funding/support provider(s) have a financial interest in the outcome of the research? Yes

3.2.2... Describe the interest.
Bulk Nutrients supply the supplement d-aspartic acid to their customers. Research which is testing the effectiveness of this supplement, can help drive up sales of the product, especially if the research provides evidence which indicates that the supplement is effective. On the other hand if the evidence from the current research indicates that it is not effective, it gives bulk nutrients the information to decide if their money is better spent elsewhere.

Bulk nutrients will be provided with ongoing updates of the current research as it unfolds. In no way will the outcome of the research be dictated by input from bulk nutrients.

3.2.2... Do you consider the funding/support arrangement constitutes:
[X] no ethical issue

3.2.2... Provide an explanation.
The product sought is a supplement which is a certified product sold by bulk nutrients, fit for human consumption. Bulk nutrients has no proprietary rights to the supplement and thus the outcome(s) of the research is available to all suppliers of the supplement.

3.2.3 Does any member of the research team have any affiliation with the provider(s) of funding/support, or a financial interest in the outcome of the research? No

3.2.4 Does any other individual or organisation have an interest in the outcome of this research No

3.2.5 Are there any restrictions on the publication of results from this research? Yes

3.2.5... Describe these restrictions.

Only group averages and standard deviations will be presented in any publication that arises from this research.

Information about individual participants will not be identifiable.

4. PRIOR REVIEWS

4.1. Ethical review

4.1.0. Duration and location

4.1.0... In how many Australian sites, or site types, will the research be conducted? 1

4.1.0... In how many overseas sites, or site types, will the research be conducted? 0

Provide the following information for each site or site type (Australian and overseas, if applicable) at which the research is to be conducted

4.1.0...Site / Site Type 1

4.1.0... Site / Site Type Name
University of Western Sydney

4.1.0... Site / Site Type Location
School of Science and Health (Campbelltown Campus, Building 20).

4.1.0...Provide the start and finish dates for the whole of the study including data analysis

Anticipated start date 01/04/2013

Anticipated finish date 09/01/2015

4.1.0... Are there any time-critical aspects of the research project of which an HREC should be aware? No

4.1.1 To how many Australian HRECs (representing site organisations or the researcher's / investigator's organisation) is it intended that this research proposal be submitted? 1

4.1.1...HREC 1

4.1.1... Name of HREC University of Western Sydney Human Research Ethics Committee (EC00314)

4.1.1...Provide the start and finish dates for the research for which this HREC is providing ethical review.

Anticipated start date or date range 01/04/2013

Anticipated finish date or date range 09/01/2015

4.1.1... For how many sites at which the research is to be conducted will this HREC provide ethical review? 1

4.1.1...Site 1

4.1.1... Name of site University of Western Sydney

4.1.1... Which of the researchers / investigators involved in this project will conduct the research at this site?

Principal Researcher(s)	Associate Researcher(s)
Mr Geoffrey Melville	
Dr Paul Marshall	
Dr Jason Siegler	

4.1.2 Have you previously submitted an application, whether in NEAF of otherwise, for ethical review of this research project to any other HRECs? No

4.3. Peer review

4.3.1 Has the research proposal, including design, methodology and evaluation undergone, or will it undergo, a peer review process? NS 1.2 Yes

4.3.1... Provide details of the review and the outcome. A copy of the letter / notification, where available, should be attached to this application.
The review of research merit for this application has been reviewed as part of Geoffrey Melville's Confirmation of Candidature (COC), which was completed on the 17th January 2013.

Ethical Review Section

Summary

Applicant / Principal Researcher(s)

Dr Paul Marshall

PhD Exercise Physiology
PG Dip Sci Exercise Physiology
BSc Sport and Exercise Science
BCom Management and Employment Relations

Over 30 peer reviewed publications specializing in exercise physiology

16 years clinical experience of exercise prescription

Supervision of approximately 40 Hons and PG Dip Sci Research Students

Potential conflicts of interest
There are no competing interests

Mr Geoffrey Melville

Bachelor of Applied Science (Sport & Exercise Science) (Hons)

Potential conflicts of interest
There are no general competing interests

Dr Jason Siegler

Dr Jason Siegler has extensive experience in study development, subject recruitment, data collection and analysis. Dr. Siegler has conducted and published independent research on many aspects of physiology, sport nutrition and performance. He has a Ph.D. in Exercise Physiology and has extensive experience in exercise testing of athletes and other populations. He is a trained phlebotomist and holds certifications in Advanced Cardiac Life Support (ACLS) and Basic Life Support (BLS).

Potential conflicts of interest
There are no competing interests

Other Relevant Personnel

Dr Paul Marshall

PhD Exercise Physiology
PG Dip Sci Exercise Physiology
BSc Sport and Exercise Science
BCom Management and Employment Relations

Over 30 peer reviewed publications specializing in exercise physiology

16 years clinical experience of exercise prescription

Supervision of approximately 40 Hons and PG Dip Sci

Research Students

Dr Jason Siegler

Dr Jason Siegler has extensive experience in study development, subject recruitment, data collection and analysis. Dr. Siegler has conducted and published independent research on many aspects of physiology, sport nutrition and performance. He has a Ph.D. in Exercise Physiology and has extensive experience in exercise testing of athletes and other populations. He is a trained phlebotomist and holds certifications in Advanced Cardiac Life Support (ACLS) and Basic Life Support (BLS).

5. PROJECT

5.1. Type of Research

5.1.1 Tick as many of the following 'types of research' as apply to this project. Your answers will assist HRECs in considering your proposal. A tick in some of these boxes will generate additional questions relevant to your proposal (mainly because the National Statement requires additional ethical matters to be considered), which will appear in Section 9 of NEAF.

This project involves:

[X] Research using quantitative methods, population level data or databanks, e.g survey research, epidemiological research NS 3.2
[X] Clinical research NS 3.3
[X] Research involving the collection and / or use of human samples NS 3.4

5.1.2 Does the research involve limited disclosure to participants? NS 2.3 — Yes

5.1.3 Are the applicants asking the HREC / review body to waive the requirement of consent? NS 2.3.5 — No

5.2. Research plan

5.2.1 Describe the theoretical, empirical and/or conceptual basis, and background evidence, for the research proposal, eg. previous studies, anecdotal evidence, review of literature, prior observation, laboratory or animal studies (4000 character limit). NS 1.1

The nutritional supplement industry is recommending DAA as a viable product to raise basal levels of testosterone. Recent research indicates that daily supplementation (3g per day) of d-aspartic acid (DAA) has the potential to increase resting levels of testosterone in untrained male participants (1). Research has demonstrated that raising levels of testosterone from exogenous administration of testosterone has a significant effect on adaptation from a training stimulus. This has not been studied with the administration of DAA. Anabolic hormones that can contribute to hypertrophy or strength gains include testosterone, insulin like growth factor 1 (IGF-1) and human growth hormone (GH). IGF-1 can increase due to resistance training (2, 3), but research has also shown non-significant changes (4-6). Basal levels of growth hormone tend not to change from resistance training (2, 6-8). This may be attributed to the large variety in isoforms produced in the body and the limited availability to detect them with current technology. Of all the hormones in the body testosterone is considered to be one of the most powerful androgenic-anabolic hormones secreted naturally by the body (9).

Evidence for the hypertrophic effects of exogenously increasing anabolic hormones has existed anecdotally for some time and research indicates these hypertrophic effects are true in normal men (10). In elderly people testosterone enanthate has been shown to improve fasting net protein balance (11), via a significant decrease in muscle protein breakdown. Testosterone has also been associated with fibre hypertrophy or more specifically the addition of myonuclei to the muscle type II fibers (12). In healthy men when testosterone concentrations were suppressed over 10 weeks, there was a reduction in an index of whole body protein synthesis (-13%), and all subjects had a decrease in fat-free mass (13).
Testosterone is also believed to stimulate the proliferation and differentiation of satellite cells (SC) (14). This has been demonstrated in young (15) and old (16) people. Satellite cells are an important mechanism of hypertrophy as they are activated by myotrauma caused by resistance training. Then the SC proliferate, move to the damaged region, where they fuse to the damaged myofiber and are eventually integrated resulting in hypertrophy (17).

It is well known that resistance exercise training leads to hypertrophy of skeletal muscle. This growth is a result of an increase in cross sectional area (CSA) and total volume of the muscle. Increased force output is a product of increased CSA in the muscle and neural adaptation (18, 19). The interaction of testosterone with ARs is not limited to muscle cells, as ARs can also be found on motor neurons. Data on gerbil studies indicates that testosterone interacting with these AR may maintain the relative size of the motor neurons (14) throughout normal life. When coupled with a resistance training program, the effect on strength and hypertrophy is enhanced (10). Testosterone administration has also been positively correlated with leg press strength, leg power and IGF levels (20). The increase in testosterone from exogenous administration of 600mg testosterone enanthate ranges from 600%-700%, which is considered supraphysiological levels (10). This is significantly greater than an expected increase from DAA.

Study 1 will measure testosterone levels before and after 12 days of DAA supplemented daily. Study 2 will

investigate the effects of the elevation of testosterone and any subsequent effects on training outcomes following a resistance exercise program. Training outcomes measured will be changes in strength hypertrophy and adaptation of the peripheral somatic nervous system.

Consequently, this thesis will be based upon novel research that strives to study the effectiveness of DAA both for its potential to raise testosterone levels and the effect on training outcomes in a resistance trained population.

5.2.2 State the aims of the research and the research question and/or hypotheses, where appropriate.

Research Aim

To measure the effect that d-aspartic supplementation has on basal testosterone levels in advanced trainers.

Primary Objectives

1. To measure testosterone levels changes.
2. To measure the change in 1RM strength
3. To measure the change in muscle CSA
4. To measure adaptation of the peripheral somatic nervous system
5. To explore the correlation of strength to other outcome measures.

4.3 Hypothesis

H1: Supplementation of 6g of DAA will elevate basal testosterone levels more than 3g of DAA as compared to the placebo.

H2: The greatest elevation in testosterone will result in greater gains in 1RM strength, which will be explained by hypertrophy of the CSA of the quadriceps muscle and adaptation of the peripheral somatic nervous system.

5.2.3 Has this project been undertaken previously? No

5.3. Benefits/Risks

5.3.0 Does the research involve a practice or intervention which is an alternative to a standard practice or intervention? No

5.3.2 What expected benefits (if any) will this research have for the wider community?

This research will provide information to the various people using d-aspartic acid (DAA) on the effectiveness of DAA to raise testosterone levels, along with informing these people of the extent to which this will help with their training goals. The proposed research will help clarify the effectiveness of DAA as a supplement for improved gains in strength as moderated by elevated levels of testosterone. Furthermore the current research will potentially open pathways for future research to explore the use of this supplement in other cohorts (elderly).

5.3.3 What expected benefits (if any) will this research have for participants? NS 2.1

All participants will receive expert advice in exercise and training. All participants should expect to receive improvements in physical strength and hypertrophy regardless of being assigned to placebo or experimental groups. Experimental groups will be given d-aspartic acid supplement at no cost for the duration of the study. All groups will receive post-workout nutrition.

5.3.4 Are there any risks to participants as a result of participation in this research project? NS 2.1 Yes

5.3.5 Explain how the likely benefit of the research justifies the risks of harm or discomfort to participants. NS 1.6

The resistance exercise intervention has been designed and will be supervised by a Geoffrey Melville (qualifications noted in section 2).Testing procedures are routine and standardised and include physical and screening assessments.

These assessments will be delivered by Geoffrey Melville and have been used across a range of cohorts. The current project involves healthy men who are at an advanced training level. Therefore, for the purposes of ethical review, this study should be considered high risk. In the case of an emergency or adverse events related to the resistance training program, emergency services (i.e. ambulance) will be notified and/or the participant will be referred to a qualified professional for follow up, as indicated. Additional risks of this study involve a small amount of discomfort experienced during blood collection, and some discomfort during neural adaptation testing methodologies.

The subjects will be informed of all steps of all the parts of the protocol as it progress's to ensure that the stress and anxiety of being a participant in this study is minimised.

As with any product consumed or brought into contact with humans, there is a risk of developing an allergic reaction. During the study all participants will be asked to consume a drink of maltodextrin (sugar) and whey protein isolate (dairy protein) for postworkout nutrition. Therefore anyone with a known allergy to these products is instantly excluded from the study. During the study participants assigned to the experimental groups will be consuming the supplement d-aspartic acid. If you have a known allergy to this product or other amino acids you will also be excluded from the study.

Human research into the product d-aspartic acid is still in early stages and thus all of the potential risks associated with consumption are unknown. Known side-effects of illegal testosterone supplementation (e.g. anabolic steroids) include increased estradiol, reduced HDL cholesterol (good cholesterol) acne, hair loss, gynecomastia, liver damage, mood changes and atrophy of the testicles. While it is thought that d-aspartic acid is likely to raise basal testosterone, current evidence from humans does not suggest the elevation will be to a level associated with reported side-effects of anabolic steroid use. Anecdotal reports of side-effects associated with d-aspartic acid use must be weighed against the likelihood people are also using a variety of other substances that may induce these side-effects. We do not expect any side-effects to be experienced (e.g. reduced HDL cholesterol, increased acne or increased estradiol). However, we will be monitoring levels of estradiol, HDL cholesterol and unwanted physical changes. Furthermore, if participants notice or experience any physical changes that they are not used to, or do not expect, they are to contact Geoffrey Melville or any of the research team as soon as possible.

Exogenous administration of testosterone to healthy males can decrease levels of HDL cholesterol. While it is hard to predict the potential health risks, it has been speculated that if this reduction in HDL cholesterol was maintained over a period of years the risk of CAD in some men might increase by about 20%. For study 2, HDL cholesterol will be analysed and monitored as a precautionary measure at baseline, 3 months and 6 months.

Participants will be reminded that they are free to withdraw at any point.

5.3.8 Are there any other risks involved in this research? eg. to the research team, the organisation, others Yes

5.3.8... What are these risks?

The monitoring of basal testosterone and HDL cholesterol can only be achieved via obtaining whole blood. Measuring blood in this manner (capillary and/or venous) is also essential for determining a) the magnitude of hormonal alteration and b) the relationships to be determined between change in testosterone and hypertrophy, strength and neural adaptation.

The collection of blood involves the use of needles and lancets. All sharps carry a risk of injury as a sharp, by definition, is anything that can cut or pierce your skin. Potentially there could also be blood borne viruses in the blood sample.

5.3.8... Explain how these risks will be negated/minimised/managed.

However, the risk of exposure to such viruses and needle-stick injury will be minimised by following the SOP for capillary and venous blood sampling (see SOPs attached) and appropriate safety measures (e.g. wearing of exam gloves, glasses and lab coat).

Once blood is obtained in vacutainer it will be centrifuged to obtain serum. The serum will be aliquoted into epindorf tubes and stored in -80oC freezer until all samples are acquired. Vacutainer method provides less chance of blood spilling, reducing the risk of exposure.

5.3.8... Explain how these risks will be monitored.

Compliance with this policy will be monitored through the annual audit process in School of Science and Health (SSH) and overseen by the Health & Safety Officer. It will be the day to day responsibility of all SSH staff members to monitor that the requirements of this procedure are being adhered too, and that appropriate risk control measures are in place. SSH staff members are responsible for ensuring those affected have followed the reporting and management procedures highlighted in the following policy: SOP_0018 Handling Accidental Inoculation Incidents.

5.3.8... Explain how any harm to participants, resulting from these risks, will be reported.

Immediate report to the injury to the senior technician, Technical Manager and Health & Safety Office (OH&S);

Complete the University's Incident Report Form and follow the OH&S policy and procedures for Accident, Injury, Incident, Hazard Reporting and Investigation.

If the source of the contamination is known, arrangements should be made to collect a blood sample from the source by the team looking after that source subject, (preferably not the injured staff member), after they have given informed written consent.

Inform the Occupational Health service immediately of the incident, so that a risk assessment can be undertaken and if necessary post-exposure prophylaxis can begin within 1 hour or as soon as possible. If the inoculation occurs outside of normal working hours, attend Accident and Emergency Department for a further risk assessment.

5.3.9 Is it anticipated that the research will lead to commercial benefit for the investigator(s) and or the research sponsor(s)? No

5.3.11 Is there a risk that the dissemination of results could cause harm of any kind to individual participants - whether their physical, psychological, spiritual, emotional, social or financial well-being, or to their employability or professional relationships - or to their communities? No

5.4. Monitoring

Refer to NS 3.3.19 - 3.3.25

5.4.1 What mechanisms do the researchers / investigators intend to implement to monitor the conduct and progress of the research project? NS 5.5

1) Reports from researchers.
The student will prepare a fortnightly summary of the progress of the research project for the fortnightly meetings with Dr. Paul Marshall and Dr. Jason Siegler.

2) Adverse incidence reports.
Any undesirable incidences will be immediately recorded and reported by the student to Dr. Paul Marshall.

3) Random inspection of research site and data.
Once the data collection phase has commenced, research investigators will perform random inspections of the research site to ensure that quality of the research procedures are being maintained. This will include inspection of the consent forms to ensure every participant being tested has a signed consent form. Data will be checked for security.

4) Participant feedback.
Participants will be advised in the participant information sheet that feedback or questions to the research investigators' are encouraged at any time.

5.4.2 Please detail your Data and Safety Monitoring Board (DSMB) and its nominee for this trial. NS 3.3.20(c)

N/A

6. PARTICIPANTS

6.1. Research participants

6.1.1 The National Statement identifies the need to pay additional attention to ethical issues associated with research involving certain specific populations.

This question aims to assist you and the HREC to identify and address ethical issues that are likely to arise in your research, if its design will include one or more of these populations. Further, the National Statement recognizes the cultural diversity of Australia's population and the importance of respect for that diversity in the recruitment and involvement of participants. Your answer to this question will guide you to additional questions (if any) relevant to the participants in your study.

6.1.1 Tick as many of the following 'types of research participants' who will be included because of the project design, or their inclusion is probable, given the diversity of Australia's population. If none apply, please indicate this below.

	c) Design specifically excludes
Women who are pregnant and the human foetus NS 4.1	[X]
Children and/or young people (ie. <18 years) NS 4.2	[X]
People highly dependent on medical care NS 4.4	[X]

6.2. Participant description

6.2.1 How many participant groups are involved in this research project? 3

6.2.2 What is the expected total number of participants in this project at all sites?
30

6.2.3. Group 1

6.2.3... Group name for participants in this group
D0

6.2.3... Expected number of participants in this group
10

6.2.3... Age range
18-36

6.2.3... Other relevant characteristics of this participant group
Inclusion criteria: Men aged between 18 and 36 with two or more years resistance training experience, who have the ability to

-squat 130% of their bodyweight and
-bench 100% of their bodyweight
-communicate in English
-no acute or chronic medical conditions which would make resistance training hazardous
-willingness and cognitive ability to provide written informed consent to participate in the trial.

6.2.3... Why are these characteristics relevant to the aims of the project?
Population target is men due to exploring the effect of d-aspartic acid, which is known to act directly on the testes to stimulate the production of testosterone.

Age restriction has been chosen because natural testosterone levels peak within this age bracket.

Training restriction has been chosen because:
-We want to explore 'true' gains from the supplementation, which in a novice population could be washed out due to the quick gains novices usually get from resistance training.
-Target market for this supplement is men who have training regularly, or who need a competitive edge.

6.2.3. Group 2

6.2.3... Group name for participants in this group
D3

6.2.3... Expected number of participants in this group
10

6.2.3... Age range
18-36

6.2.3... Other relevant characteristics of this participant group
Inclusion criteria: Men with two or more years resistance training experience, who have the ability to

-squat 130% of their bodyweight and
-bench 100% of their bodyweight
-communicate in English
-no acute or chronic medical conditions which would make resistance training hazardous
-willingness and cognitive ability to provide written informed consent to participate in the trial.

6.2.3... Why are these characteristics relevant to the aims of the project?
Population target is men due to exploring the effect of d-aspartic acid, which is known to act directly on the testes to stimulate the production of testosterone.

Age restriction has been chosen because natural testosterone levels peak within this age bracket.

Training restriction has been chosen because:
-We want to explore 'true' gains from the supplementation, which in a novice population could be washed out due to the quick gains novices usually get from resistance training.
-Target market for this supplement is men who have training regularly, or who need a competitive edge.

6.2.3. Group 3

6.2.3... Group name for participants in this group
D6

6.2.3... Expected number of participants in this group
10

6.2.3... Age range
18-36

6.2.3... Other relevant characteristics of this participant group
Inclusion criteria: Men with two or more years resistance training experience, who have the ability to

-squat 130% of their bodyweight and
-bench 100% of their bodyweight
-communicate in English
-no acute or chronic medical conditions which would make resistance training hazardous
-willingness and cognitive ability to provide written informed consent to participate in the trial.

6.2.3... Why are these characteristics relevant to the aims of the project?
Population target is men due to exploring the effect of d-aspartic acid, which is known to act directly on the testes to stimulate the production of testosterone.

Age restriction has been chosen because natural testosterone levels peak within this age bracket.

Training restriction has been chosen because:
-We want to explore 'true' gains from the supplementation, which in a novice population could be washed out due to the quick gains novices usually get from resistance training.
-Target market for this supplement is men who have training regularly, or who need a competitive edge.

6.2.4. Your response to questions at Section 6.1 - Research Participants indicates that the following participant groups are excluded from your research. If this is not correct please return to section 6.1 to amend your answer.

Children and/or young people (ie. <18 years)

Women who are pregnant and the human foetus

People highly dependent on medical care

6.2.4... Have any particular potential participants or groups of participants been excluded from this research? In answering this question you need to consider if it would be unjust to exclude these potential participants. NS 1.4
Children and young people have been excluded as such individuals will unlikely have average and stable testosterone levels, and also at a training level required for the study.

Pregnant women will be excluded as the pregancy will have direct and confounding effect on the

physiological outcomes of this trial. Women in general have been excluded as the physiological mechanism for which d-aspartic acid mainly works is via increased secretion of testosterone in the testes.

People highly dependent on medical care will not be able to perform resistance exercise without direct supervision, which cannot occur within a group-type environment.

6.3. Participation experience

6.3.1 Provide a concise detailed description, in not more than 200 words, in terms which are easily understood by the lay reader of what the participation will involve.

Study 1 will involve 2 weeks standardised training before the experimental period begins. Baseline testing will commence at the end of week 2 (involving 1 repetition max testing and a venous blood-draw). Participants will be randomised to one of three groups and begin their daily allocated dose (0g 3g or 6g) and continue training for a further 12 days. After 12 days a post blood draw will be taken along with strength testing.

Study 2 will involve 12 laboratory testing sessions, where strength (1RM, neural adaptation (electrical nerve stimulation), hypertrophy (ultrasound) and testosterone (venous blood) will be measured. For the duration of the study participants will be consuming capsules daily either containing placebo or a testosterone boosting supplement. They will also be attending the University of Western Sydney research gym four times per week for the 6 month duration of the study. Post workout nutrition will be provided in liquid form at the end of each session.

For more information please refer to attached participation information sheet –study 1 and participation information sheet study 2.

6.4. Relationship of researchers / investigators to participants

6.4.1 Specify the nature of any existing relationship or one likely to rise during the research, between the potential participants and any member of the research team or an organisation involved in the research.

Geoff Melville is a PhD student involved in teaching of some units through the Sport and Exercise Science Program, and therefore may be in contact with potential participants who are enrolled in related courses.

Dr. Paul Marshall is the coordinator for several courses run through the Sport and Exercise Science Program, therefore he may be in contact with potential participants who are enrolled in related courses.

Dr. Jason Siegler is the director of academic programming for the Sport and Exercise Science Program, therefore he may be in contact with potential participants who are enrolled in related courses.

6.4.2 Describe what steps, if any, will be taken to ensure that the relationship does not impair participants' free and voluntary consent and participation in the project.

If there are any students of the researchers, they will be informed prior to providing informed consent that Geoff/Paul/Jason are investigators in this research, and that their potential participation in this study will not affect their academic studies or relationship with any of the three researchers in any way.

6.4.3 Describe what steps, if any, will be taken to ensure that decisions about participation in the research do not impair any existing or foreseeable future relationship between participants and researcher / investigator or organisations.

Professional communication will be demonstrated at all times.

If there are any students of the researchers, they will be informed prior to providing informed consent that Geoff/Paul/Jason are investigators in this research, and that their potential participation in this study will not affect their academic studies or relationship with any of the three researchers in any way.

6.4.4 Will the research impact upon, or change, an existing relationship between participants and researcher / investigator or organisations.? No

6.5. Recruitment

6.5.1 What processes will be used to identify potential participants?

Advertisements will be placed on notice boards around the University of Western Sydney, Campbelltown campus. There will also be online advertising at the UWS website using the vUWS function, which places notices on the homepage of students.

Additionally, advertisements will be placed in local recreational training facilities (with consultation of the manager of the facility).

Finally, a newspaper advertisement will be purchased to help attract participants from the local community. Potential participants will be invited to contact the investigators directly via email or phone,at which point

they will be sent a Participant Information Sheet to consider their participation in the study.

6.5.2 Is it proposed to 'screen' or assess the suitability of the potential participants for the study? Yes

6.5.2... How will this be done?

Pre-screening will be conducted via PAR-Q and a standardised health history questionnaire. Moderate risk participants will require approval of their physician prior to participation. High risk participants will be excluded

All selection criteria will be outlined in the participant information sheet (See attached).

6.5.3 Describe how initial contact will be made with potential participants.

Potential participants will contact the researcher via email or phone in response to adverts and flyers.

Initial contact will be made via email, the email will provide an invitation to the study. If there is interest in the study another email will be sent with a copy of the participant information sheet and consent form. If further clarification is required, contact is made in person or over the phone.

Potential participants are offered the opportunity to ask questions before consenting to participate. Testing will not commence until informed written consent to participate is obtained by the researchers

6.5.3... Do you intend to include both males and females in this study? No

6.5.3... Please explain why only one sex is involved in the study. In doing this you will need to demonstrate why this approach is valid.

The main outcome measure for the proposed study is testosterone levels. The supplement d-aspartic acid is known to increase testosterone levels by accumulating in the testes and up-regulating transportation of cholesterol which is a limiting factor in the rate of testosterone production. Currently there has been no research conducted on the effects of d-aspartic acid on testosterone levels in women.

6.5.4 Is an advertisement, e-mail, website, letter or telephone call proposed as the form of initial contact with potential participants? Yes

6.5.4... Provide details and a copy of text/script.

(This is a sample participant recruitment email)

Dear "Potential Participant",

My name is Geoff Melville and I am a PhD candidate at the University of
Western Sydney. I would like to extend an invitation to you, inviting you to participate in a research project I am conducting titled:

The effect of a testosterone boosting supplement on adaptation from a 24 week resistance training program?

Please find below/attached an information document detailing your role as a potential participant in this project. You may register your interest in participating in this research project with myself (Geoff Melville – Principle Researcher) where you will be forwarded a participant information sheet for you to decide if you wish to participate in this research. Testing is expected to be underway in April 2013.

Participants involved in this project will undergo 2 weeks of standardised training. Baseline testing will commence at the end of this week. Participants will be randomised to their group, either 3g Supplement(S3), 6g Supp (S6) or placebo (S0) then begin their daily allocated dose and resistance training program for 12 weeks. After training for 12 weeks both groups will have a one week break (de-load week) to help prevent overtraining, during this period groups will also stop taking d-asp/placebo pills, they will then continue training for another 12 weeks and start taking the d-asp/placebo again. Eight blood draws will be taken in total. These will be conducted at baseline, 12 days, 30 days, 90 days, second baseline and repeated measures at the same time points will follow for the next 12w cycle.

Phase two will involve 2 groups, S0 and experimental group S. Participants will conduct 12 weeks of training, a one week break, then another 12 weeks of training, with the experimental group consuming DAA daily for the whole 25 weeks. Strength and neural testing will be conducted pre, mid and post training.

I would like to thank you for considering this invitation.
Kind regards,

Geoff Melville (AEP)
PhD Candidate, Exercise Science

BHSc(Hons), Sport and Exercise Science
School of Science and Health

Mobile: +614 2626 3 496
Office: (02) 4620 3917
B.20.Fax (02) 4620 3020
Email: g.melville@uws.edu.au

Office Address:
Building 20 G Room 35
School of Science and Health
UWS Campbelltown Campus
Narellan Road, CAMPBELLTOWN NSW 2560

Work Postal Address:
Building 20.G.35
School of Science and Health
UWS Campbelltown Campus
Locked Bag 1797
PENRITH SOUTH DC NSW 1797

6.5.5 If it became known that a person was recruited to, participated in, or was excluded from the research, would that knowledge expose the person to any disadvantage or risk? No

6.6. Consent process

6.6.1 Will consent for participation in this research be sought from all participants? Yes

6.6.1... Will there be participants who have capacity to give consent for themselves? Yes

6.6.1... What mechanisms/assessments/tools are to be used, if any, to determine each of these participant's capacity to decide whether or not to participate?

The PIS and CF are tools that will help us determine whether or not participants comprehend the written description of the study. In addition, we will be providing a verbal description of the study to participants (in person or over phone), and will provide opportunities for potential participants to ask questions relating to any aspect of the study that they do not understand.

6.6.1... Are any of the participants children or young people? No

6.6.1... Will there be participants who do not have capacity to give consent for themselves? No

6.6.1... Describe the consent process, ie how participants or those deciding for them will be informed about, and choose whether or not to participate in, the project.

Consent will be received in written format from potential participants after the initial contact process and information about the study (PIS) is provided to them. Furthermore, potential participants will be pre-screened via procedures outlined in the previous section with the PAR-Q and Health Screening Questionnaire prior to providing informed consent.

The consent process (including information provided) depends on the method of initial contact with the researchers. If contact is made in person a brief description of the study will be verbally provided. Here it will be determined whether the potential participant is interested in receiving further information about the study.If so, contact details will be asked for so that we are able to send the PAR-Q, health screening form, as well as the participant information sheet (PIS) and consent form (CF).

If contact is made via phone, again a brief description of the study will be provided and if the individual is interested a copy of the above forms may be sent to them via mail. If by email a reply email will be sent with the appropriate forms (in PDF format) attached. No matter how contact is made potential participants are offered the opportunity to ask questions before consenting to participate. Testing will not commence until the PAR-Q and health screening are completed, and informed written consent to participate is obtained by the researchers. Regardless of the means of communication, potential participants will be offered several opportunities to ask questions before consenting to participate, thereby ensuring they fully understand what is required of them.

6.6.1... If a participant or person on behalf of a participant chooses not to participate, are there specific consequences of which they should be made aware, prior to making this decision? 4.6.6 - 4.6.7

There are no consequences for choosing not to participate. If a participant discloses that they take performance enhancing supplements, this will only exclude them from the study and this information will not be disclosed to any other third party.

6.6.1... Might individual participants be identifiable by other members of their group, and if so could this identification expose them to risks?

Yes it is possible for participants to identify each other. It is possible that multiple participants will be training in the gym at the same time. This, however, will not expose any participant to any greater risk. At no point will personal information or results be disclosed to anyone other the specific participant the results relate to.

6.6.1... If a participant or person on behalf of a participant chooses to withdraw from the research, are there specific consequences of which they should be made aware, prior to giving consent?

There are no consequences for choosing to withdraw at any point in the study.

6.6.1... Specify the nature and value of any proposed incentive/payment (eg. movie tickets, food vouchers) or reimbursement (eg travel expenses) to participants.

There are no proposed material incentives/payments or reimbursements.

6.6.1... Explain why this offer will not impair the voluntary nature of the consent, whether by participants' or persons deciding for their behalf. NS 2.2.10 - 2.2.11

As there are no proposed material incentives/payments or reimbursements, there are no offers that will effect the voluntary nature of consent.

6.6.3 Do you propose to obtain consent from individual participants for your use of their stored data/samples for this research project? No

6.6.3... Give justification

We will not be using any previously collected or stored data in this study.

8. CONFIDENTIALITY/PRIVACY

8.1. Do privacy guidelines need to be applied in the ethical review of this proposal?

8.1.1 Indicate whether the source of the information about participants which will be used in this research project will involve:

[X] collection directly from the participant

8.1.1... Information which will be collected for this research project directly from the participant

8.1.1... Describe the information that will be collected directly from participants. Be specific where appropriate.

Age, height, weight, date of birth, street address, email address, emergency contact details, telephone number.

Relevant medical history (pre exercise health screening questionnaire).

Strength levels during a squat and bench press exercise

Size of the quadriceps muscles (thigh) using non-invasive doppler ultrasound imaging techniques.

Muscle activation of the quadriceps muscles using surface electromyography.

Neural adaptation to resistance training via changes in evoked v-wave and h-reflex responses.

Changes in testosterone will be measured via a venipuncture collection of a blood sample taken in the morning after a 12h fast. Fingerprick samples of blood will also be collected for the measurement of HDL cholesterol.

8.1.1... The information collected by the research team about participants will be in the following form(s). Tick more than one box if applicable.

[X] individually identifiable
[X] non-identifiable

8.1.1... Give reasons why it is necessary to collect information in individually identifiable or re-identifiable form.

The individual data will be in identifiable form during the intervention study owing to the repeated measures performed on each participant, therefore appropriate data matching is required to the participant's previous results.

Upon completion of the intervention, data will be de-identified.

Data may be used for further publications or investigations in the future.

Participant identities will remain confidential, with only group averages and percentages being presented.

Therefore, it will not be possible for individual participants to be identified in any publication of this study.

The PIS will provide participants information on how their data will be used, and more importantly, will be protected.

8.1.1... Consent process

You have indicated that you will be varying the conditions of or waiving consent. See questions in section 6.6

8.1.1... Will consent be specific or extended or unspecified? NS 2.2.14 - 2.2.18 Specific

8.1.1... Provide reasons why this form of consent has been chosen. You may need to revise your answer at 6.6.1.1.3 to provide details on the consent process

Data collected during this research project will only be used for the purposes of this project and publications based on this project.

8.2. Using information from participants

8.2.1 Describe how information collected about participants will be used in this project.

Dependent variables will be analysed with appropriate data and statistical analysis procedures. Analysis methods to be used will be decided upon by Geoff Melville as part of their PhD.

While group averages for the dependent variables may be used in future research publications and conferences, it will not be possible to identify any participant's results.

8.2.2 Will any of the information used by the research team be in identified or re-identifiable (coded) form? Yes

8.2.2... Indicate whichever of the following applies to this project:

[X] Information collected for, used in, or generated by, this project will/may be used for another purpose by the researcher for which ethical approval will be sought.

8.2.4 List ALL research personnel and others who, for the purposes of this research, will have authority to use or have access to the information and describe the nature of the use or access. Examples of others are: student supervisors, research monitors, pharmaceutical company monitors .

Information will only be in an identifiable form during the experimental period. Following completion of data collection and providing an individual result summary to participants', all data will be completely de-identified.

Geoff Melville
Dr Paul Marshall
Dr Jason Siegler

8.3. Storage of information about participants during and after completion of the project

8.3.1 In what formats will the information be stored during and after the research project? (eg. paper copy, computer file on floppy disk or CD, audio tape, videotape, film)

- Paper copy
- Computer files

8.3.2 Specify the measures to be taken to ensure the security of information from misuse, loss, or unauthorised access while stored during and after the research project? (eg. will identifiers be removed and at what stage? Will the information be physically stored in a locked cabinet?)

All paper date and information will be archived for a period of 5 years in a locked file cabinet in the research laboratory of building 20 in the Sport and Exercise Science Program, UWS Campbelltown.

All computer files and electronic data records will be saved on password protected servers within the Sport and Exercise Science Program, UWS Campbelltown. Only research investigators will be able to access computer data files.

After the data collection period is complete, computer data will be de-identified.

Given the this research involves a proposed waiver of consent and the intent of exposing illegal activity [see NS 4.6.1] the HREC must be satisfied that your response to this question has justified that there is sufficient protection of the privacy of the participants.

8.3.5 The information which will be stored at the completion of this project is of the following type(s). Tick more than one box if applicable.

[X] non-identifiable

8.3.6 For how long will the information be stored after the completion of the project and why has this period been chosen?

Information will be stored for the mandatory 5 year period of data storage.

8.3.7 What arrangements are in place with regard to the storage of the information collected for, used in, or generated by this project in the event that the principal researcher / investigator ceases to be engaged at the current organisation?

All paper data will remain locked in a file cabinet in the research laboratory of building 20 in the Sport and Exercise Science Program, UWS Campbelltown, for a period of 5-years.

Electronic data will be stored on the protected server for the Sport and Exercise Science Program. Only the research investigators will have access to these files.

If the research investigators cease to be engaged with the current organisation, arrangements will be made with the Sport and Exercise Science Technical Staff Officer to delete all electronic files and shred and dispose of all hard-copy information at the end of the 5-year period.

8.4. Ownership of the information collected during the research project and resulting from the research project

8.4.2 Who is understood to own the information resulting from the research, eg. the final report or published form of the results?

There are no third parties with ownership rights over this research. In accordance with the UWS Intellectual Property Policy – Part E – (22) in accordance with legal principles, the University, as employer, is the owner

of intellectual property created by staff, in the course of their employment.In accordance with conventions in the university community for many years, the University will not claim ownership of copyright in scholarly works. A student will own copyright subsisting in the student's thesis.

The University will not claim ownership of copyright in a student thesis
Publication of the results in a peer reviewed journal will see a transfer of the copyright to the publishers of the final article. In this instance, the publisher will not have access to any information which could personally identify any participant in the study.

8.4.3 Does the owner of the information or any other party have any right to impose limitations or conditions on the publication of the results of this project? No

8.5. Disposal of the information

8.5.1 Will the information collected for, used in, or generated by this project be disposed of at some stage? Yes

8.5.1... At what stage will the information be disposed?
Information will be destroyed after the mandatory 5 year storage period

8.5.1... How will information, in all forms, be disposed?
Paper data will be shredded and computer files will be deleted.

8.6. Reporting individual results to participants and others

8.6.1 Is it intended that results of the research that relate to a specific participant be reported to that participant? Yes

8.6.1... Specify in what form the results will be reported to participants.
Email correspondence if available, otherwise a hard copy will be posted to participants.

8.6.1... How will the results be communicated to participants? eg telephone call, individual letter, copy of publication, consultation with a medical practitioner or other
Results will be communicated to the participants via email or letter, with an attachment of their individual results, as well as group averages.

8.6.1... Who will be responsible for communicating the project results to participants?
Geoff Melville

8.6.2 Is the research likely to produce information of personal significance to individual participants? Yes

8.6.3 Will individual participant's results be recorded with their personal records? No

8.6.4 Is it intended that results that relate to a specific participant be reported to anyone other than that participant? No

8.6.5 Is the research likely to reveal a significant risk to the health or well being of persons other than the participant, eg family members, colleagues No

8.6.6 Is there a risk that the dissemination of results could cause harm of any kind to individual participants - whether their physical, psychological, spiritual, emotional, social or financial well-being, or to their employability or professional relationships - or to their communities? No

8.6.7 How is it intended to disseminate the results of the research? eg report, publication, thesis
Journal publications (group averages only).

Individual data will not be able to be identified in any dissemination of these results.

8.6.8 Will the confidentiality of participants and their data be protected in the dissemination of research results? Yes

8.6.8... Explain how confidentiality of participants and their data will be protected in the dissemination of research results
Once data collection is complete, individual results will be deidentified, thereby ensuring participant identities will remain confidential. Furthermore, only group averages and percentages will be presented in any publication of the results from this study.

9. PROJECT SPECIFIC

9.1. Type of research/trial

**9.1.1 The administration of a drug / medicine (includes a complementary / alternative medicine)**	Yes
**9.1.2 The use of a medical device**	No
**9.1.3 The administration of human somatic cell gene therapy**	No
**9.1.4 The use of a xenotransplant**	No
**9.1.5 The use of stem cells (adult or embryonic) as therapy**	No
**9.1.6 Other**	No

9.1.7. The project will be conducted as follows:

**9.1.7... Under the Clinical Trial Notification Scheme (CTN)**	No
**9.1.7... Under the Clinical Trial Exemption Scheme (CTX)**	No

You have indicated that you are conducting a clinical trial under neither the CTN or CTX scheme. Please ensure that this is correct by referring back to your answer at Q 5.1.1.2. If you are conducting a trial in a clinical setting, which will not take place under CTN or CTX, please ensure that enough detail has been provided about the research to allow a HREC to adequately review it. This may require you to review your answers in section 5.1.1 and/or 6.1.1.

9.1.8. Provide the following details for the clinical trial protocol:

9.1.8... Protocol name
NA

9.1.8... Protocol version number
NA

9.1.8... Protocol version date 07/02/2013

9.1.8... If you intend to/have registered this trial in a publicly accessible register, please provide the details of it here NS 3.3.12
NA

9.1.9. Provide the following details for the investigator's brochure/product information (as relevant):

9.1.9... Title of Investigator's Brochure
NA

9.1.9... Investigator's brochure version number
NA

9.1.9... Investigator's brochure version date 07/02/2013

9.2. Clinical research

9.2.1. The study examines:

**9.2.1... The administration of a drug / medicine (includes a complementary / alternative medicine)**	No
**9.2.1... The use of a medical device**	No
**9.2.1... Other**	Yes

4.1.1... Describe briefly the type of study to be conducted
Investigation of the sports supplement d-aspartic acid

9.2.2. Provide the following details for the study protocol:

**Protocol title**	NA
**Protocol version number**	NA

Protocol version date 07/02/2013

9.2.3. Provide a statement describing the following:

9.2.3... Method of randomisation

Random group allocation using the random block generator program available at www.random.org

9.2.3... Whether the hypothesis offers a realistic possibility that the intervention is at least as effective as standard treatment.

Supplementation of d-aspartic acid is expected to be more effective than exercise alone.

9.2.3... The justification for the use of placebo or non-treatment control group, including alternative effective treatments and any risk of harm in the absence of treatment.

There is no risk of harm for the absence of the supplement in the placebo group.

9.2.3... How variations in response will be treated

Individual progression during the program will be monitored by the assessments at the end of every 2-weeks.

Training loads will be adjusted for each individual based on assessment results.

9.2.3... Endpoints

Study 1.
2 weeks of standardised training and 2 weeks supplementation period (1 month)

Study 2.
25 weeks of training and supplementation (6 month)

9.2.3... Details of contingencies and management of these

Direct supervision of training once per week and monitoring of training and nutritional diaries will allow management of each participant during the training period.

9.2.3... Explain the arrangements in place to ensure there is adequate compensation for participants.

No financial compensation is arranged.

9.2.4 How many drugs will be used in this research project? 0

9.5. Research involving the collection and / or use of human samples

You have indicated that the project involves the use of human samples.

9.5.1 What is the nature of sample/s you plan to use?

Whole blood

-Venipuncture and vacutainer samples   Hormonal analysis
-Capillary samples   HDL Cholesterol analysis

9.5.2 What is the source of the sample/s you wish to use? (tick all boxes that apply)

[X] Collected from participants recruited to this research project who are not concurrently undergoing diagnosis or treatment.

9.5.2... By whom will the sample/s be collected? Please detail the process of collection. NS 3.4.1 (a-f)

A member of the research team

9.5.3 In what form will the sample(s) be used by the investigators in the conduct of this project? Potentially identifiable (ie coded)

9.5.4 Will the tissue sample(s) used for this project be destroyed once the project is completed? Yes

9.5.5 Does this research involve the development of a cell line? No

9.5.6 Provide details of the collection and management of this information source. NS 3.4.1 (a-f)

The collection of blood has been outlined in three standard operating procedures pertaining to venous blood sampling (SOP0016), capillary blood sampling (SOP0019) and procedures for handling accidental inoculations incidents (SOP0018) previously approved by the Biosafety and Radiation Safety Committee (BRSC) and School of Science and Health (SoH).

All paper date and information will be archived for a period of 5 years in a locked file cabinet in the Sport and Exercise Science Program, UWS Campbelltown.

All computer files and electronic data records will be saved on password protected servers within the Sport and Exercise Science Program, UWS Campbelltown.

Only research investigators will be able to access computer data files.
After the data collection period is complete, computer data will be de-identified.

9.5.7 Describe how you will ensure that all sample/s used in this project will be stored securely and describe how you will monitor this as well as the use of the sample/s.

Once blood is obtained in vacutainer it will be centrifuged to obtain serum. The serum will be aliquoted into epindorf tubes and stored in -80c freezer until all samples are acquired.

The capillary samples obtained will be analysed immediately upon collection in a desktop analyser (Reflotron). Once analysed (~ 2 min), the samples will be discarded into appropriate waste/hazard containers.

10. DECLARATIONS AND SIGNATURES

10.1 Project Title

The effect of D-Aspartic Acid on adaptation from a 24 week resistance training program

10.2 Human Research Ethics Committee to which this application is made

University of Western Sydney Human Research Ethics Committee (EC00314)

10.3 Signatures and undertakings

Applicant / Principal Researchers (including students where permitted)

I/we certify that:
- All information is truthful and as complete as possible.
- I/we have had access to and read the National Statement on Ethical Conduct in Research Involving Humans.
- the research will be conducted in accordance with the National Statement.
- the research will be conducted in accordance with the ethical and research arrangements of the organisations involved.
- I/we have consulted any relevant legislation and regulations, and the research will be conducted in accordance with these.
- I/we will immediately report to the HREC anything which might warrant review of the ethical approval of the proposal NS 5.5.3 including:
  - serious or unexpected adverse effects on participants;
  - proposed changes in the protocol; and
  - unforseen events that might affect continued ethical acceptability of the project.
- I/we will inform the HREC, giving reasons, if the research project is discontinued before the expected date of completion NS 5.5.6 see NS 5.5.8(b);
- I/we will adhere to the conditions of approval stipulated by the HREC and will cooperate with HREC monitoring requirements. At a minimum annual progress reports and a final report will be provided to the HREC.

Applicant / Chief Researcher(s) / Principal Researcher(s)

Dr Paul Marshall ________________________ ____/____/______
University of Western Sydney Signature Date

Mr Geoffrey Melville ________________________ ____/____/______
University of Western Sydney Signature Date

Dr Jason Siegler ________________________ ____/____/______
University of Western Sydney Signature Date

Supervisor(s) of student(s)

I/we certify that:
- I/we will provide appropriate supervision to the student to ensure that the project is undertaken in accordance with the undertakings above;
- I/we will ensure that training is provided necessary to enable the project to be undertaken skilfully and ethically.

Dr Paul Marshall ________________________ ____/____/______
Signature Date

Dr Jason Siegler ________________________ ____/____/______
Signature Date

Heads of departments/schools/research organisation

I/we certify that:
- I/we are familiar with this project and endorse its undertaking;

- the resources required to undertake this project are available;
- the researchers have the skill and expertise to undertake this project appropriately or will undergo appropriate training as specified in this application.

________ Title	________________ First name	____________________________ Surname
____________________________ Position		____________________________ Organisation name
____/____/____ Date		____________________________ Signature

11. ATTACHMENTS

This page and all pages that follow don't need to be submitted to your HREC.

11.1 List of Attachments

**Core Attachments**	**Attachments which may be required/appropriate.**
Recruitment/invitation	Copy of advertisement, letter of invitation etc
Participant Information	Copy or script for participant<br>Copy or script for parent, legal guardian or person responsible as appropriate
Consent Form	Copy for participant<br>For parent, legal guardian or person responsible as appropriate<br>For, optional components of the project eg. genetic sub study
Peer review	Copy of peer review report or grant submission outcome
HREC approvals	Copy of outcome of other HREC reviews

**Attachments specific to project or participant group**	**Attachments which may be required/appropriate.**
Epidemiological research	Evidence of support/permission from database custodian for proposed access / use of data
Administration of a drug for research but is not clinical research	Technical information about the drug
Clinical trial under CTN/CTX scheme	Outcome of TGA evaluation under CTX scheme<br>Compensation statement<br>Indemnity agreement<br>Insurance certificate<br>Clinical trial agreement<br>TGA forms for signature if study approved
Research involving the collection and / or use of human samples	Evidence of support/permission from tissue bank or tissue custodian for proposed access / use of tissue<br>Evidence of having met any legal requirements for use of autopsy or coronial autopsy tissue
Research involving assisted reproductive technologies (ART)	Evidence of having met any legal requirements relevant to the research
Children and/or young people (ie. <18 years)	Information/consent form for parent, legal guardian or person responsible
People highly dependent on medical care	Information/consent form for legal guardian or person responsible

11.2 Participant information elements
Core Elements
Provision of information to participants about the following topics should be considered for all research projects.

Core Elements	Issues to consider in participant information
About the project	Full title and / or short title of the project<br>Plain language description of the project<br>Purpose / aim of the project and research methods as appropriate<br>Demands, risks, inconveniences, discomforts of participation in the project<br>Outcomes and benefits of the project<br>Project start, finish, duration
About the investigators / organisation	Researchers conducting the project (including whether student researchers are involved)<br>Organisations which are involved / responsible<br>Organistions which have given approvals<br>Relationship between researchers and particpants and organisations
Participant description	How and why participants are chosen<br>How participants are recruited<br>How many participants are to be recruited
Participant experience	What will happen to the particant, what will they have to do, what will they experience?<br>Benefits to individual, community, and contribution to knowledge<br>Risks to individual, community<br>Consequences of participation
Participant options	Alternatives to participation<br>Whether participation may be for part of project or only for whole of project<br>Whether any of the following will be provided: counselling, post research follow-up, or post research access to services, equipment or goods
Participants rights and responsibilities	That participation is voluntary<br>That participants can withdraw, how to withdraw and what consequences may follow<br>Expectations on participants, consequences of non-compliance with the protocol<br>How to seek more information<br>How to raise a concern or make a complaint
Handling of information	How information will be accessed, collected, used, stored, and to whom data will be disclosed<br>Can participants withdraw their information, how, when<br>Confidentiality of information<br>Ownership of information<br>Subsequent use of information<br>Storage and disposal of information
Unlawful conduct	Whether researcher has any obligations to report unlawful conduct of participant
Financial issues	How the project is funded<br>Declaration of any duality of interests<br>Conspensation entitlements<br>Costs to participants<br>Payments, reimbursements to participants<br>Commercial application of results
Results	What will particpants be told, when and by whom<br>Will individual results be provided<br>What are the consequences of being told or not being told the results of

Core Elements	Issues to consider in participant information
	research<br>How will results be reported / published<br>Ownership of intellectual property and commercial benefits
Cessation	Circumstances under which the participation of an individual might cease<br>Circumstances under which the project might be terminated

Research Specific Elements
Provision of information to participants about the following topics should be considered as may be relevant to the research project.

Specific to project or participant group	Additional issues to consider in participant information
Administration of a drug for research but is not clinical research	Information about the drug, contra-indications etc
Clinical trial under CTN/CTX scheme	Explain that the trial therapy is experimental<br>Describe the phase of the trial, where applicable<br>Describe arms of trial, where applicable and explain what the trial therapy is being compared to eg. standard therapy or placebo<br>Describe the meaning of placebo, randomisation, cross-over, wash-out period etc as applicable<br>Compensation and indemnity arrangements as applicable / appropriate
Research involving the collection and / or use of human samples	How samples will be accessed, collected, used, stored, and disposed of<br>Who will have access to samples<br>Can participants withdraw their samples, how, when<br>Ownership of samples<br>Subsequent use of samples including development of cell lines<br>Any legal requirements / constraints on collection or use of samples eg. autopsy, coronial autopsy<br>Any legal requirements regarding consent for use of sampels eg. autopsy, coronial autopsy
Research involving assisted reproductive technologies (ART)	Have the consent requirements of the NHMRC ART guidelines (pg 50) been met?
