# Supplementary material for: The effects of d-aspartic acid supplementation in resistance-trained men over a three month training period: A randomised controlled trial
Source: PLoS One. 2017 Aug 25;12(8):e0182630. doi: 10.1371/journal.pone.0182630 (PMC5571970; doi:10.1371/journal.pone.0182630)
Supplement: S3 File — (DOCX) [file pone.0182630.s003.docx]

Participant Information Sheet

# Project Title: Exploring the effectiveness of D-Aspartic Acid on training adaptation during a 12-week training program

## Who is carrying out the study?

You are invited to participate in a study conducted by PhD candidate Geoffrey Melville (School of Science and Health), under the supervision of Dr Paul Marshall and Dr Jason Siegler.

## What is the study about?

The purpose of this study is to investigate the effect of d-aspartic acid has on changes in fasted basal testosterone levels from 3 months of training. D-aspartic acid is a naturally occurring, endogenous amino acid that is found in nervous and endocrine tissues of the human body. Previous research has suggested that d-aspartic acid has the potential to increase levels of testosterone. The supplement d-aspartic acid will be sourced from supplier Bulk Nutrients and is fit for human consumption. This study will investigate the effects that elevation of testosterone has on training outcomes following a resistance exercise program. Training outcomes measured will be changes in strength, hypertrophy and adaptation of the peripheral somatic nervous system.

## Is there an eligibility criteria?

Two or more years resistance training experience (consistent training excluding de-loading phases); male; 18 – 36 years old; healthy; have the ability to squat at least 130% of their bodyweight; have the ability to bench 100% of their bodyweight; ability to communicate in English; no acute or chronic medical conditions which would make resistance training hazardous or primary outcome measures impossible to assess; willingness and cognitive ability to provide written informed consent to participate in the trial.

As with any product consumed or brought into contact with humans, there is a risk of developing an allergic reaction. During the study all participants will be asked to consume a drink of maltodextrin (sugar) and whey protein isolate (dairy protein) for post workout nutrition. Therefore anyone with a known allergy to these products is instantly excluded from the study. During the study participants assigned to the experimental groups will be consuming the supplement d-aspartic acid. If you have a known allergy to this product or other amino acids you will also be excluded from the study.

## What does the study involve?

The study will require you to attend the research laboratory at UWS Campbelltown campus building 20 for three testing time points (which may be 1-2 sessions long depending on time available), and conduct training at the university research gym on multiple occasions over a 3 month period. The first session will involve testing 1 repetition maximum (1RM) for the back squat and the bench press. Initial testing will also involve measurement of the thigh muscle using ultrasound technology and measurement of the peripheral somatic nervous system using electrical nerve stimulation. Blood draws will be taken at 0 weeks, 6 weeks and 3 months. During blood draws participants will be required to come in to the lab in the morning (7-10 am) after a 12 hour fast (food, coffee). After a 5-10 minute resting period, the investigator will take a venous blood sample (3-4 tubes) from the forearm (similar to a normal blood test). This will be analysed for hormonal levels (TT, FT, E2, SHBG, ALB) and HDL cholesterol. For the three days surrounding the blood draw testing day (i.e. day before/day of/day after) participants are required to fill out a 24h food diary (9 total).

For the duration of the study participants will come in for supervised training sessions. The first week participants are expected to come in for the four days to become familiar with each of the training days. From then on participants will continue to training four times per week, and are only required to come in for one supervised session, the other three conducted at their normal gym. At the supervised sessions participants will be asked to bring in the provided training diary for photocopying (to allow for ongoing data entry).

During the experimental period participants will consume a dose of pills (11 capsules) containing the supplement (DAA) or a placebo each morning. They will train for 12 weeks, with small changes in the program at the 6 week mark. Participants will also be expected to consume the provided post workout nutrition within 30 minutes of finishing their workout.

## How much time will the study take?

The study will be conducted over approximately 3 months. Your involvement will require you to attend the research laboratory or research gym on the previously mentioned occasions. The first session for each time point will involve 1RM testing of the bench press and squat, and provided criteria is met this will be followed by neural testing of the calf muscle. This will take approximately 30-45 minutes for 1RM testing and 90 minutes for the calf protocol. The second session for each time point will involve venous blood draws followed by ultrasound of the quad and calf. Blood draw takes approximately 20 minutes (allowing for relaxation period) and the ultrasound will take 30-75 minutes, varying from person to person. There is the option if the participate prefers to complete testing in one day. If this was the case the order of testing would be bloods, ultrasound, 1RMs, and neural stimulation. Furthermore they would be asked to bring in something they could consume for breakfast to have after the bloods and ultrasound been completed. Bear in mind this could take 3-4.5 hours.

## Will the study benefit me?

At the end of the study you will be given access to the results of the study. This will provide you with information about the effectiveness of this particular supplement for you as an individual. Throughout the study post-workout nutrition in the form of powder will be provided free of charge. You’re weekly allocation of post workout nutrition will be provided to you when you come to your weekly supervised session. Over 3 months of professional, motivated training, participants should expect to see gains in strength and/or hypertrophy.

## Will the study involve any discomfort for me?

Yes. First the 1RM testing sessions are designed to find how much weight you are able to move for one repetition, which can be uncomfortable. Second, the sampling of venous blood involves inserting a sharp needle into one of your forearm veins (like a standard blood test). Most people experience this as a brief, transient sharp pain. Third, nerve stimulation involves a brief electrical stimulus applied to a nerve in your leg. Some experience this as a sharp, but transient pain. Fourth, the amount of training volume required for the study is designed to match as close as possible the average volume for an advanced trained population, therefore you may experience some discomfort from Delayed Onset of Muscle Soreness (DOMS).

## Are there any risks?

Human research into the product d-aspartic acid is still in early stages and thus all of the potential risks associated with consumption are unknown.  Known side-effects of testosterone supplementation (e.g. anabolic steroids) include increased estradiol, reduced HDL cholesterol (good cholesterol) acne, hair loss, gynecomastia, liver damage, mood changes and atrophy of the testicles. While there is evidence to suggest that d-aspartic acid can raise basal testosterone, current evidence from humans does not suggest the elevation will be to a level associated with reported side-effects of anabolic steroid use. Anecdotal reports of side-effects associated with d-aspartic acid use must be weighed against the likelihood people are also using a variety of other substances that may induce these side-effects.  We do not expect any side-effects to be experienced (e.g. reduced HDL cholesterol, increased acne or increased estradiol).  However, we will be monitoring levels of estradiol, HDL cholesterol and unwanted physical changes. Furthermore, if you notice or experience any physical changes that you are not used to, or do not expect, please contact Geoffrey Melville or any of the research team as soon as possible.

## How is this study being paid for?

The study is being sponsored by Higher Research Degree funds available to PhD candidates, for the School of Science and Health at University of Western Sydney. Supplement sponsorship is provided by Bulk Nutrients, TAS, Australia.

## Will anyone else know the results? How will the results be disseminated?

All aspects of the study, including results, will be confidential and only researchers will have access to information on participants. Data collected from you will be presented in undergraduate classes, research seminars, conference presentations, postgraduate theses and/or research publications; under none of these circumstances will your identity be revealed to the audience or the readership. Information collected for this project, or generated by this project may be used for another purpose by the researcher, for which ethical approval will be sought. It may be possible for participants to identify each other. Potentially there may be multiple participants training in the gym at the same time. This, however, will not expose any participant to any greater risk. At no point will personal information or results be disclosed to anyone other the specific participant the results relate to.

## Can I withdraw from the study?

Participation is entirely voluntary. You are not obliged to be involved and, if you do participate, you can withdraw at any time without giving any reason and without any consequences.

## Can I tell other people about the study?

Yes, you can tell other people about the study by providing them with the chief investigator’s contact details. They can contact the chief investigator to discuss their participation in the research project and obtain an information sheet.

## What if I require further information?

When you have read this information sheet, PhD candidate Geoff Melville will discuss it with you and answer any questions you might have. If you would like to know more at any stage, please feel free to contact Geoff Melville [g.melville@uws.edu.au](mailto:g.melville@uws.edu.au), 0426263496, 46203917

## What if I have a complaint?

This study has been approved by the University of Western Sydney Human Research Ethics Committee (HREC). The approval number is H10087.

If you have any complaints or reservations about the ethical conduct of this research, you may contact the Ethics Committee through the Office of Research Services on Tel +61 2 4736 0229 Fax +61 2 4736 0013 or email humanethics@uws.edu.au.

Any issues you raise will be treated in confidence and investigated fully, and you will be informed of the outcome. If you agree to participate in this study, you may be asked to sign the Participant Consent Form.
